# Supplementation with short-chain fatty acids and a prebiotic improves clinical outcome in Parkinson’s disease: a randomized double-blind prospective study

**DOI:** 10.1038/s41598-025-29692-x

**Published:** 2025-12-05

**Authors:** Tobias Hegelmaier, Alexander Duscha, Christiane Desel, Sabrina Fuchs, Michal Shapira, Sivan Amidror, Qihao Shan, Gabriele I. Stangl, Frank Hirche, Stefan Kempa, András Maifeld, Lisa-Marie Würtele, Jana Peplinski, Diana Jauk, Gitali Naim, Nuphar Shidlovsky, Adva Cohen, Yifat Bennet, Lisa Paschold, Claudia A. Dumitru, Ute Obermüller-Jevic, Svein-Olaf Hustvedt, Nina Timmesfeld, Ralf Gold, Antonia Zapf, Mascha Binder, Ibrahim E. Sandalcioglu, Sanaz Mostaghim, Horst Przuntek, Eran Segal, Nissan Yissachar, Aiden Haghikia

**Affiliations:** 1 Department of Neurology, Otto-Von-Guericke University, Magdeburg, Germany; 2 Department of Neurology and Clinical Neurophysiology, Hannover Medical School, OE 7210, Carl-Neuberg-Str. 1, 30625 Hannover, Germany; 3 German Center for Neurodegenerative Diseases (DZNE), Magdeburg, Germany; 4 Department of Molecular Cell Biology, Weizmann Institute of Science, Rehovot, Israel; 5 Department of Computer Science and Applied Mathematics, Weizmann Institute of Science, Rehovot, Israel; 6 The Mina and Everard Goodman Faculty of Life Sciences, Bar-Ilan University, Ramat-Gan, Israel; 7 Bar-Ilan Institute of Nanotechnology and Advanced Materials, Bar-Ilan University, Ramat-Gan, Israel; 8 Faculty of Computer Science, Otto-Von-Guericke University Magdeburg, Magdeburg, Germany; 9 Institute of Agricultural and Nutritional Sciences, Martin-Luther-University Halle-Wittenberg, Halle (Saale), Germany; 10 Integrative Metabolomics and Proteomics, Berlin Institute of Medical Systems Biology/Max Delbrück Center for Molecular Medicine, Berlin, Germany; 11 Department of Neurology, Ruhr University Bochum, St. Josef Hospital Bochum, Bochum, Germany; 12 Internal Medicine IV, Oncology/Hematology, Martin-Luther-University Halle-Wittenberg, Halle (Saale), Germany; 13 Department for Neurosurgery, Otto-Von-Guericke University, Magdeburg, Germany; 14 Nutrition & Health Division, GBU Nutrition Ingredients, BASF SE, Ludwigshafen, Germany; 15 Nutrition & Health Division, GBU Pharma Solutions, BASF A/S, Oslo, Norway; 16 Department for Medical Informatics, Biometry and Epidemiology, Ruhr-University, Bochum, Germany; 17 Institute of Medical Biometry and Epidemiology, University Medical Center Hamburg-Eppendorf, Hamburg, Germany; 18 Department of Biomedicine, University of Basel, Basel, Switzerland; 19 Clinic of Neurology II, EVK Hattingen, Hattingen, Germany

**Keywords:** Parkinson’s disease, Gut microbiome, Immunomodulation, Short chain fatty acids, Clinical improvement, Neurodegeneration, Neuroinflammation, Immunology, Microbiology, Neuroimmunology

## Abstract

**Background:**

Parkinson’s disease is associated with a dysbiotic, proinflammatory gut microbiome, disruptions to intestinal barrier functions, and immunological imbalance. Microbiota-produced short-chain fatty acids, such as propionic and butyric acid promote gut barrier integrity and immune regulation, but their impact on Parkinson’s disease pathology remains mostly unknown.

**Methods:**

In a randomized double-blind prospective study, 72 people with Parkinson’s disease received propionic and butyric acid and/or the prebiotic fiber 2′-fucosyllactose supplementation over 6 months in combination with existing Parkinson’s disease-specific therapy. Patients underwent complete neurological assessment and provided blood and stool samples before as well as 3 and 6 months after supplementation.

**Results:**

We observed a robust improvement in motor symptoms, with all intervention groups achieving clinically meaningful reductions. These motor benefits were paralleled by clinically relevant reductions in levodopa medication. In contrast, effects on nonmotor symptoms were more heterogeneous. Notably, the interventions also modulated peripheral immune responses and enhanced mitochondrial respiration in immunocytes. Postintervention microbiota remodeled inflammatory and barrier-related gene sets in gut organ cultures and improved in vitro barrier functions. Treatment response was associated with microbiome composition, distinct patterns of colonic transcription and permeability ex vivo. Multiobjective analysis revealed immune parameters associated with an optimal response to supplementation.

**Conclusion:**

Short-chain fatty acids ameliorate clinical symptoms in Parkinson’s disease patients and modulate intestinal and peripheral immunity.

Registration: This clinical trial was retrospectively registered with the German Clinical Trials Register (DRKS), registration number DRKS00027061 on 11/19/2021.

**Supplementary Information:**

The online version contains supplementary material available at 10.1038/s41598-025-29692-x.

## Introduction

Parkinson’s disease (PD) is one of the most common progressive systemic neurodegenerative disorders, affecting millions of people worldwide. Despite intensive research, the cause of neurodegeneration is not fully understood. The current state of research assumes a multifactorial etiology. In addition to sporadic forms of genetic predisposition, mitochondrial dysfunction, oxidative stress and neuroinflammation, also environmental factors play a crucial role^1,2^. Gastrointestinal symptoms are often the first nonmotor symptoms in PD, in addition to olfactory dysfunction, which in most cases occur years to decades before the first motor symptoms, i.e., rigor, tremor and akinesis^3^. PD incidence rates are rising along with changes in nutrition and the consumption of Western-style diets, which are low in fiber and high in saturated fats and refined carbohydrates. These dietary habits lead to a dysbiotic intestinal microbiota and altered metabolome as well as intestinal inflammation^4,5^. Growing evidence supports the idea that microbial dysbiosis, leaky gut syndrome and a proinflammatory intestinal environment are central components of the pathogenesis of PD and may affect the response to therapeutic interventions^6–11^.

The gut metabolome is essential for metabolic homeostasis but is also involved in communication between the microbiome and the subepithelial structures of the gut, which has multifold systemic implications for the organism. Short-chain fatty acids (SCFAs) are a major group of metabolites involved in the microbiome-gut interaction and are produced through the anaerobic fermentation of dietary fibers. A large proportion of SCFAs are metabolized by colonocytes or in the liver, where they contribute to the energy supply^12^. However, a broad range of intracellular SCFA effects are also mediated by G-protein-coupled receptors and SCFA transporters. These include maintenance of intestinal barrier integrity, mucus production, protection against intestinal and systemic inflammation and blood‒brain barrier (BBB) permeability^13^. In PD patients, the levels of SCFA-producing bacteria and fecal SCFAs are significantly reduced^14^. In a recent study investigating the potential therapeutic effect of propionic acid (PA), we observed a putative neuroprotective effect in addition to immune regulation^15^. Targeting the gut-brain axis via the microbiome, metabolome and intestinal barrier is a promising approach for future therapies for PD. Consumption of prebiotic fibers has recently been tested in a small cohort of PD patients over 10 days^16^. However, SCFA supplementation in PD patients over a prolonged period has not yet been evaluated.

We conducted a randomized, double-blind prospective study over 6 months to investigate direct supplementation of the SCFAs PA and butyric acid (BA) as well as the prebiotic 2′-fucosyllactose (2FL), which induces the synthesis of both nutritional compounds (PA and BA)^17,18^. In order to determine the impact of supplementation on microbiome diversity and colonic gene expression, we used shotgun metagenomic sequencing of collected stool samples and took advantage of an in vitro 3D gut organ culture model. We performed in-depth immunophenotyping and T-cell receptor (TCR) sequencing before and after 6 months of supplementation. In addition, we recruited a verification cohort to further expand on the impact of SCFA supplementation on the function of regulatory T cells (Treg), and performed in vitro co-culture experiments. Last, we devised a model to predict whether the response to supplementation was affected by the patients’ microbiome, and employed multiobjective analysis (MOA) to incorporate the various clinical measurements and thus more accurately determine the response to intervention. The primary endpoints of this study were the impact on microbiome diversity and composition as well as changes of SCFA concentration in stool and serum. Because the study was exploratory, no correction for multiplicity and no confirmatory statements are made. The secondary endpoints were the effect on the clinical parameters defined by The Movement Disorder Society-Sponsored Revision of the Unified Parkinson’s Disease Rating Scale (MDS-UPDRS III), levodopa equivalent daily dose (LEDD), Parkinson Neuropsychometric Dementia Assessment (PANDA), and olfactory score. We identified novel mechanistic links between SCFA supplementation, modulation of intestinal responses and systemic immunity, and clinical outcomes in PD patients. This likely involves mucosal-associated invariant T cell (MAIT)-mediated mechanisms, with recently described potential neuroprotective effects^19^.

## Methods

### Study design and analysis

The study was performed from November 2019 to August 2020 after being approved by the Ethics Committee of the Ruhr-University Bochum (November 2019; registration number 19–6713). The study visits were unaffected by the SARS-CoV-2 pandemic; however, an impact on physical activity and physical therapy is presumed. Prior to participation, all subjects signed informed consent forms. This clinical trial was retrospectively registered in the German Clinical Trials Register (DRKS; registration number DRKS00027061). Group specific sample size was calculated by a priori power analysis with fixed effects. Based on previous studies with SCFA in patients with Multiple Sclerosis, effect size was set to 0.4 with standard 0.05 α error and a power of 0.85 for three investigation groups. Power calculation resulted a total sample size of 72 individuals with an actual power of 0.8534928. A total of 94 participants were assessed for eligibility and 72 were confirmed for randomization and assigned to one treatment group upon recruitment. Participants were eligible if they were between 18 and 90 years of age, diagnosed with a primary Parkinsonian syndrome, had a moderate disease severity and were fully oriented. Exclusion criteria included immunosuppressive therapy within the past 6 months (e.g., mitoxantrone, azathioprine, cyclophosphamide, methotrexate), Exclusion criteria included immunosuppressive therapy within the past 6 months (e.g., mitoxantrone, azathioprine, cyclophosphamide, methotrexate), the use of antibiotics or metformin in the past three months because of their effects on metabolism and the gut microbiome^20^, and strict vegan dietary habits.

Randomization was performed by permuted block randomization to one of the three study arms. The randomization list was prepared by BASF SE (Baden Aniline and Soda Factory, Societas Europaea) and provided to the hospital pharmacy in a sealed envelope. All patients were instructed to take either 3600 mg PA + BA capsules (BA: 2400 mg; PA: 1200 mg) with 3900 mg placebo, 3900 mg 2FL capsules with 3600 mg placebo or 3900 mg 2FL capsules with 3600 mg PA + BA capsules daily for up to 6 months in combination with existing PD-specific therapy. The PA + BA, 2FL and placebo were provided as delayed-release capsules by BASF SE. Blood and fecal samples were collected at the Department of Neurology, St. Josef Hospital Bochum, at the Clinic of Neurology II, EVK Hattingen, and at the University Clinic of Neurology, UK Magdeburg. Clinical data were evaluated by 2-way ANOVA with time and treatment as factors or mixed effects analysis with patient as random effect. It was not corrected for multiplicity, so the p-values are used as descriptive measures. Fecal samples were immediately frozen at  −80 °C. Serum tubes were centrifuged at 30 min after the blood draw, and the supernatant frozen at  −80 °C. For isolation of peripheral blood mononuclear cells (PBMC), blood was drawn in EDTA tubes and separated using Cytiva Ficoll-Paque™ PLUS. Isolated cells were frozen in CTL-Cryo medium (Immunospot) and stored at  −80 °C.

### Metagenomic sequencing and bioinformatics analysis

Metagenomic DNA was extracted using the DNeasy PowerMag Soil DNA extraction kit (Qiagen), which was optimized for the Tecan automated platform. Next-generation sequencing (NGS) libraries were prepared using Illumina’s Nextera DNA library prep and sequenced on an Illumina NovaSeq sequencing platform with 100 bp single-end reads at a depth of 10 million reads per sample. Reads containing Illumina adapters and low-quality reads were filtered out, and the ends of low-quality reads were trimmed. To eliminate host DNA contamination, reads were mapped to the human genome using bowtie with inclusive parameters, and matches were discarded. The relative abundance of bacterial species was obtained by an expanded microbial genome reference recently published by the Segal lab, with default parameters^21^. Microbiome ∝-diversity was calculated by the Shannon diversity index. Richness was calculated as the number of species in the sample detected with an abundance of at least 1e-4. All abundances were logarithmically transformed. Comparisons between microbial relative abundances and indices were performed using the Mann‒Whitney U test. To evaluate the discriminative power of microbial composition for R\NR prediction, we developed an XGBoost^22^ prediction model that exclusively utilizes microbiome features as inputs. This model can effectively capture nonlinear interactions between bacteria and has been previously demonstrated to outperform other methods for human microbiome data classification^23^. The receiver operating characteristic (ROC) curves mean and standard deviation were calculated using the curves produced in a fivefold cross-validation approach. To ensure the model’s robustness, we conducted a label-swapping analysis to determine that the performance of the model was no better than random prediction, resulting in an area under the curve (AUC) value very close to 0.5. To gain insights into the model’s interpretability, we utilized SHAP (SHapley Additive explanation)^24^ to analyze feature attributes. The SHAP values represent the average change in the model’s output when conditioning on a specific feature.

### Quantification of SCFA levels in serum samples

The method of serum preparation was adapted from McArthur and Sarnaik^25^. Two hundred microliters of serum and 20 µl of internal standard were mixed. Afterward, 200 µl of this mixture was added to 10 µl of 70% perchloric acid and 20 µl of 2 M hydrochloric acid, and extraction was performed by adding 1 ml of cold diethyl ether. After phase separation, 800 µl of the organic phase was vortexed with 7.5 µl of 4 M sodium hydroxide. The sample was dried first in a stream of liquid nitrogen at room temperature and subsequently at 60 °C. The residue was solubilized in 20 µl of 2 M hydrochloric acid and diluted with isopropanol to a final volume of 400 µl. After centrifugation at 200 × g for 3 min, SCFA levels were measured by HPLC‒MS/MS. For HPLC‒MS/MS measurements, an Agilent 1100 HPLC with a Poroshell HPH-C18 column (150 mm × 2.1 mm, 4 mm, Agilent Technologies) and an API 2000 (Applied Biosystems) were used. Elution was performed at 42 °C using a gradient of methanol + 10 mM ammonium formate pH 8 (20 + 80), methanol + water + formic acid (80 + 20 + 0.1) and methanol. After removal from the column, the eluent was acidified by methanol + water + formic acid (180 + 20 + 0.4).

### Quantification of SCFA levels in fecal samples

Analysis of SCFA levels in fecal samples was performed as previously described^26^ with modifications. In brief, 10 mg of fecal material was homogenized in extraction solution containing 100 ml of 100 mM crotonic acid (internal standard; Sigma Aldrich), 50 ml hydrochloric acid and 200 ml ether using 2.8 mm Precellys ceramic beads (Bertin Technologies). Homogenization was performed at room temperature for 10 min at 1500 rpm on a horizontal shaker unit followed by 10 min of centrifugation at 1000 × g at room temperature. Then, 80 ml of the upper ether phase was added to a fresh glass vial with 16 ml of N-tert-butyldimethylsilyl-N-methyltrifluoroacetamide (MTBSTFA, Sigma Aldrich) and incubated at 80 °C for 20 min at 500 rpm on a horizontal shaker unit. Afterward, the samples were left for 24 h at room temperature for derivatization. HPLC‒MS/MS measurements were performed as described above.

### Mice

C57BL/6 J mice were obtained from Envigo RMS (Israel), and bred in the specific-pathogen-free facility at Bar-Ilan University, Israel. All animal procedures were carried out in accordance with relevant guidelines and local regulations and according to the protocol approved by the Bar-Ilan University ethics committee (ethics approval number BIU-IL-2205–146-3). Mice were sacrificed by decapitation at 12–14 days of age and at a weight of 6–7 g. All methods were reported in accordance with the ARRIVE guidelines. All methods were reported in accordance with the ARRIVE guidelines.

### Fecal bacterial cultures

Fecal samples from PD patients were resuspended in 500 µl of sterile phosphate-buffered saline (PBS). For aerobic cultures, serial dilutions were plated on brain–heart infusion (BHI) plates and incubated at 37 °C for 24 h. For anaerobic cultures, serial dilutions were plated on BHI rich medium plates and incubated in an anaerobic chamber (Don Whitley Scientific) at 37 °C for two days. Bacterial colonies were then counted to calculate the bacterial load (CFU/gr).

### Ex vivo gut organ culture system

Gut organ culture experiments were performed as described^15,27^. Each gut organ culture experiment included 6 colons dissected from littermate mice infused with microbiota derived from one patient before and after intervention in duplicate. Two additional colon organ cultures were infused with sterile culture medium and served as an internal control. Tissues were collected for analysis at 4 h postcolonization. Fecal samples from *n* = 4 best responding (largest decrease in MDS-UPDRS III after 6 months of supplementation) and *n* = 4 nonresponding (largest increase in MDS-UPDRS III after 6 months of supplementation) patients were used.

### RNA sequencing and bioinformatics analysis

Colon tissue fragments (~ 3 mm) were placed in RNAlater overnight at 4 °C, and then the samples were stored at  −80 °C. For RNA extraction, tissues were homogenized using a bead beater, and RNA was extracted with the Qiagen RNeasy Micro kit (Qiagen). The Illumina TruSeq stranded mRNA library prep kit (Illumina) was used for library generation, and next-generation sequencing was performed at the Bar-Ilan University scientific equipment center using the Illumina NextSeq platform (NextSeq 500 High Output v2 kit). RNAseq fastq files from 24 samples from 8 patients were aligned to the *M. musculus* reference genome mm10 using STAR (version 2.7.10a)^28^. The resulting BAM files were sorted and indexed using Samtools. Read counts per gene were performed using htseq-count^29^ (version 0.12.4). Differential gene expression analysis was performed using the DESeq2 (1.36.0) R/bioconductor package. DESeq2 applies Wald’s test on normalized counts and uses a negative binomial generalized linear model, which determines differentially expressed genes and log-fold changes. Genes for which the average counts in all samples were less than 25 were filtered. The batch effect of the experiment column in the metadata was removed by the function removeBatchEffect from the limma (version 3.52.2) package in R. Significantly differentially expressed genes were selected using threshold values of *p* value ≤ 0.05 and log2fold change ≥ 0.58 (FC >  = 1.5) and/or following FDR correction (FDR p-adjusted ≤ 0.1, Benjamini‒Hochberg). Volcano plots were generated for data visualization using ggplot2 (version 3.4.0). For pathway enrichment analysis, differentially expressed genes were analyzed using Metascape^30^. Additionally, Gene Set Enrichment Analysis (GSEA)^31^ (GSEA version 4.2.3) was performed for all the genes that were ranked (-log10(pvalue)/sign(log2FoldChange)) with M5 ontology gene sets. Relevant pathways from GSEA results with q-value ≤ 0.05 were plotted using ggplot2 (version 3.4.0).

### Cell lines

The human colon colorectal adenocarcinoma (CaCo-2) cell line was kindly provided by Prof. Ohad Gal Mor (Sheba Medical Center, Israel). The cells were cultured at 37 °C and 5% CO_2_ until reaching approximately 95% confluence and were then subcultured using a trypsin-B solution. The cells were maintained in Dulbecco’s modified Eagle medium (DMEM-F12) supplemented with 20% heat inactivated fetal bovine serum (FBS), L-glutamine and 100 U/mL penicillin/streptomycin. For coculture experiments, CaCo-2 cells were cultured for 4 d and then incubated for 6 h in the presence of sterile (filtered) fecal suspensions or fecal supernatants (for epithelial adhesion assay).

#### RNA extraction and real-time PCR

RNA was extracted from CaCo-2 cells using Direct-zol™ RNA Microprep (ZYMO). The concentration and absorbance at 260 nm and 280 nm were measured to assess RNA purity. RNA was reverse transcribed into cDNA using qSqript (Quantabio). SYBR Green (Thermo Fisher Scientific) was used to evaluate gene expression using a real-time PCR apparatus (CFX 96, Bio-Rad). Relative *Tjp1* (ZO-1) expression was quantified using a real-time PCR assay with *Tjp1*-specific primers (Forward: 5′-CGGTCCTCTGAGCCTGTAAG -3′; Reverse: 5′- GGATCTACATGCGACGACAA -3′), with *Eef2* as the reference gene (Forward: 5′-AACTTCACGGTAGACCAGATCC-3′; Reverse: 5′- TCGTCCTTCCGGGTATCAGTG -3′). Relative gene expression levels were determined using the 2 − ΔΔCT method.

#### Dynamic transepithelial electrical resistance (TEER) measurements

CaCo-2 cells were plated on a CytoView Z 96-well impedance plate (3.4 × 10^5^ cells per well for 4 days) (Axion BioSystems) and monitored by the Maestro Edge platform (Axion BioSystems) until reaching full confluence (800–1200 Ω). The barrier index (which represents barrier integrity) was calculated by the Axion ‘Impedance’ module as the ratio between cellular resistance at low frequency (1 Hz) vs. high frequency (41 Hz). For coculture experiments, fecal samples were resuspended in cell culture media without antibiotics to a concentration of 3.3 mg/ml. Suspensions were centrifuged at 5000 rpm for 3 min, and supernatants were centrifuged again at 5000 rpm for 5 min. Following filtration of supernatants in a Sterile Syringe Filter 0.22 µm, a final volume of 200 μL was added to each CaCo-2-well. The barrier index was measured at a high temporal resolution of 1 min for 24 h and was normalized to the reference time (t = 0). An additional correction was performed by normalizing to the barrier index of unstimulated cells.

#### 16S-DNA sequencing of epithelial-adhesive microbes and bioinformatics analysis

CaCo-2 cells were cultured to full confluency with penicillin/streptomycin for 4 days. Fecal samples were resuspended in cell culture media without antibiotics to a concentration of 15 mg/ml. Suspensions were passed through a sterile cell strainer and then added to CaCo-2 cells. Following incubation for 30 min, cells were washed 3 times with PBS to remove nonadherent bacteria. DNA was extracted using the PureLink™ Microbiome DNA Purification Kit (Invitrogen). 16S rRNA sequencing was performed by Hylabs LTD, Israel. The QIIME pipeline (version qiime2-2022.11) was used on 16S rRNA gene raw sequences from microbial communities. The pipeline includes importing the files, demultiplexing, denoising and removing chimeras using the dada2 algorithm, creating a phylogenetic tree using the MAFFT program to align the sequences and the FastTree algorithm to build the tree. The samples were then rarefied by QIIME2 to a minimum sequence depth of 24,949 reads/sample. Alpha (Faith pd, Shannon and Observed features metrices) and beta diversity (unweighted unifrac, weighted unifrac, jaccard_distance and Bray_curtis distances) analyses were performed. Taxonomy was assigned using the classify-sklearn naïve base classifier against the GreenGenes database^32^. After the QIIME pipeline was used, the taxonomy was further filtered: nonspecific taxa identified in the sterile control samples were removed. Data were normalized (relative abundance per sample) and a paired T test from the ggpubr (version 0.6.0) package in R was used to compare two related groups (pretreatment and posttreatment samples, per patient). A comparison plot with p values less than 0.05 was plotted using GraphPad Prism 9.

#### Ex vivo gut permeability assay (X-IPA)

For quantitative analysis of gut permeability dynamics at the whole-tissue level, we developed a novel ex vivo intestinal permeability assay, X-IPA, as previously described^33^. Briefly, fluorescein isothiocyanate (FITC)-dextran (4 kD) was resuspended in sterile culture medium without phenol red and infused into the gut lumen using a syringe pump, with or without patient fecal samples (diluted to a bacterial concentration of 10^7^ CFU/ml), pre- and post-SCFA intervention. Experiments were terminated at 4 h poststimulation. At the experimental endpoint, the FITC-dextran concentration in the extraintestinal medium was measured by quantifying the fluorescence intensity using a fluorometer.

#### Immunophenotyping

For in-depth immunophenotyping archived PBMCs were quickly thawed in a 37 °C water bath before resuspension in ice-cold PBS (Gibco) and washed once. A total of 1 × 10^6^ cells were stained per panel. Cells were preincubated with human TrueStain FcX™ (BioLegend) to block nonspecific binding. Staining panels and gatings (Supplementary Tables S1 and S2) were modified based on Monaco et al.^34^. All phenotyping experiments were performed on a BD FACSCelesta™ (Beckton Dickinson) with standardized application settings and analyzed by BD FACS DIVA v9 software (Beckton Dickinson).

#### Treg suppression assay

PBMCs from whole blood of PD patients from the validation cohort (baseline and after 14 days of supplementation with 2FL or BA + PA) were isolated by Ficoll Paque PLUS (GE Healthcare) gradient centrifugation and separated via a MACS CD4^+^CD25^+^CD127^dim/-^ Regulatory T-Cell Isolation Kit II human (Miltenyi Biotec) according to the manufacturer’s protocol. Briefly, CD127^dim/-^ cells were isolated from 5 × 10^7^ PBMCs using negative enrichment and subsequently subjected to positive selection of CD25^+^ regulatory T cells. CD127^+^ and CD25^-^ cells were pooled and used as controls (PBMCs). PBMCs (2 × 10^6^) were stained with a CellTrace CFSE Cell Proliferation Kit (Thermo Scientific) to investigate proliferation in a mixed lymphocyte reaction. Then, 5 µg/ml αCD3 (UCHT1, Invitrogen) and 1 µg/ml αCD28 (CD28.2, Invitrogen) were added to induce proliferation. A total of 5 × 10^4^ cells were seeded per well in duplicate in serum-free X-VIVO™ 15 (Lonza) on 96-well plates. Tregs, as well as unstained PBMCs, were seeded at a 1:1 ratio with CFSE-stained PBMCs. As a reference stain for autologous proliferation of CFSE-stained cells, PBMCs were seeded separately without coculture. All cells were cultured at 37 °C with 5% CO_2_ for 4 days. Proliferation was analyzed by flow cytometry on an Attune NxT (Thermo Fisher Scientific). Suppressive capacity was calculated after normalization to autoproliferation. To determine the suppression of PBMCs and Tregs, their individual levels of proliferation were subtracted from the proliferation levels of CD3/CD28-stimulated PBMC-cell without coculture. Afterward, the suppressive capacity of Tregs was calculated as the ratio of PBMC suppression divided by Treg suppression.

For Treg/PBMC cocultures with in vitro addition of BA and PA, blood from healthy controls or PD patients without SCFA supplementation was used, and Treg/PBMC cells were isolated as described above and seeded on a 1:1 ratio in serum free TheraPEAK™ X-VIVO™ 15 (Lonza) without CFSE staining. Cells were stimulated with 5 g/ml PHA (Phytohemagglutinin, Merck), 150 M BA (sodium butyrate, Merck) and 150 M PA (sodium propionate, Merck) for 4 days as indicated. Cell culture supernatants were analyzed using a LEGENDplex™ Human Essential Immune Response Panel (13-plex) (BioLegend) according to the manufacturer’s protocol and recoded on a BD FACSCelesta™ cell analyzer (Beckton Dickinson).

#### qPCR analysis of sorted Tregs

For quantitative real-time PCR analysis, PBMCs from PD patients from the validation cohort were isolated by Ficoll Paque PLUS (GE Healthcare) gradient centrifugation and 5 × 10^7^ cells were stained with αCD4-FITC (RPAT4, BD Biosciences), αCD25-APC (BC69, BioLegend) and αCD127-PE (HIL-7R-M21, BD Biosciences), and CD4^+^CD25^++^CD127^lo^ Tregs were sorted on a BD FACSAria™ III (Beckton Dickinson). The yield of highly purified Tregs was typically between 2–3 × 10^5^ per sort. RNA was isolated using an RNeasy Micro kit (Qiagen). One hundred nanograms of RNA was transcribed into cDNA using a QuantiTect® Reverse Transcription Kit (Qiagen), and qPCR was performed on a QuantStudio™ 7 Real-Time PCR system using Applied Biosystems TaqMan® Gene Expression assays and Applied Biosystems TaqMan Fast Advanced Master Mix. Gene expression assays: *B2m*: Hs00187842_m1, *Ccr8*: Hs04969449_m1, *Cmc1*: Hs00976539_g1, *Crot*: Hs00221733_m1, *Ctla4*: Hs00175480_m1, *Foxp3*: Hs01085834_m1, *Il10*: Hs00961622_m1, *Il17rb*: Hs00218889_m1, *Xpa*: Hs00902270_m1 (all Thermo Fisher Scientific).

#### Seahorse XF cell mito stress test

Frozen PBMCs were thawed and seeded on sterile poly-D-lysine-coated 6-well Agilent Seahorse XF Cell Culture Microplates at a density of 3 × 10^5^ cells per well in X-VIVO™ 15 (Lonza). Cells were incubated for 24 h at 37 °C in a humidified 5% CO_2_ incubator. Sensor cartridges were hydrated overnight in Seahorse XF Calibrant medium at 37 °C without CO_2_. The cell culture medium was replaced with Seahorse medium containing DMEM, 2 mM sodium pyruvate, 10 mM glucose and 2 mM L-glutamine (all Gibco, Thermo Fisher Scientific) and incubated at 37 °C without CO_2_ for 1 h prior to Seahorse assay performance. Cellular metabolic activity was measured using a Seahorse XFp Cell Mito Stress Test Kit (Agilent Technologies) on a Seahorse XFp analyzer according to the manufacturer’s protocol. Individual parameters of basal respiration, ATP production, maximal respiration, proton leak, spare capacity and nonmitochondrial oxygen consumption were calculated according to the Seahorse XFp Cell Mito stress test protocol.

#### TCR-β sequencing

RNA was isolated from 5 × 10^6^ frozen PBMC (*n* = 8 responder, n = 8 nonresponder at baseline and after 6 months) using an RNeasy Mini kit (Qiagen) with on-column DNase treatment. RNA quality control, library preparation and TCR-β sequencing were performed by CeGAaT GmbH (Tübingen, Germany). For library preparation, 125 ng RNA and the library preparation kit AmpliSeq™ for Illumina® TCR beta-SR Panel were used. Sequencing was performed on a NovaSeq6000 (Illumina) with 2 × 100 bp reads. Demultiplexing of the sequencing reads was performed with Illumina bcl2fastq (2.20). Adapters were trimmed with Skewer (version 0.2.2)^35^. Quality trimming of the reads was not performed. Paired FASTQ files were processed, aligned and annotated for the TCR-β VDJ region using the MiXCR^36^ pipeline with the default NCBI library. Sequences with identical CDR3 nucleotide sequences were assembled as one clone and comprised a defined frequency (0–1) within the repertoire according to the summed read counts. Nonproductive TCR-β clones and clones with fewer than 2 reads were removed and data normalized to 1,000,000 TCR-β reads. After quality control, repertoires from *n* = 6 responders and *n* = 6 nonresponders at baseline as well as from *n* = 5 responders and *n* = 7 nonresponders at V2 remained, including *n* = 5 responder and *n* = 5 nonresponder matched pairs (baseline, V2). Further analysis of the TCR-β repertoire and plotting was performed using R version 4.2.1 and packages ade4^37^, ggplot2^38^ and tidyverse^39^ or GraphPad Prism version 8.3.1. Calculation of the repertoire metrics clonality, richness, Shannon and Simpson diversity indices as well as clonal space distribution and TRBV gene usage was performed using the R package tcR. For analysis of overlapping clones, the numbers of reads from the repertoires were downsampled to 200,000 reads. An overlapping clone was defined as a clone with an identical CDR3 amino acid sequence present both at baseline and V2. Clones with increasing or decreasing frequency after treatment were matched for identical CDR3 amino acid sequences with TCR-β sequences with known epitope recognition listed in the public database VDJdb^40^ (accessed at 2^nd^ August 2022).

#### Multiobjective analysis

MOA is based on the concept of multiobjective optimization and is applied to problems with several conflicting objectives, where increasing the quality in one objective results in deteriorating the others. The goal of MOA is to evaluate the data based on several such objectives and identify the underlying features explaining the variability. The central idea concerns the nondominated sorting of a dataset^41^ and then clustering the data according to the so-called fronts. Considering the clinical dataset *A* of N patients, we sort them according to the domination criterion as follows: A data point X dominates a data point Y given the objectives (metrics) f1 = PANDA, f2 = MDS-UPDRSIII and f3 = olfactory score if:

$${f}_{i}\left(X\right)\preccurlyeq {f}_{i}\left(X\right), \forall i and \exists j:{f}_{j}\left(X\right)\prec {f}_{j}(Y)$$

where < and ≤ denote better and equal or better, respectively.

By applying the domination criterion to the dataset *A*, we can identify a subset of data points that are not dominated by any other. This subset is indicated as Front 1. In the next step, we consider the dataset *A* without the data points in F1 and perform the same procedure. The remaining nondominated points are stored in a subset F2. By performing this procedure iteratively, we can sort the dataset into several fronts from F1 (best subset) to Fw (worst subset). In this way, every data point of each patient has a front number. For the analysis in this paper, we took 20% of patients with the lowest and highest front numbers and sorted them into clusters ${A}_{1}$ and ${A}_{2}$.

## Results

### Improved clinical outcome upon 6 months supplementation in PD patients

**Fig. 1:**
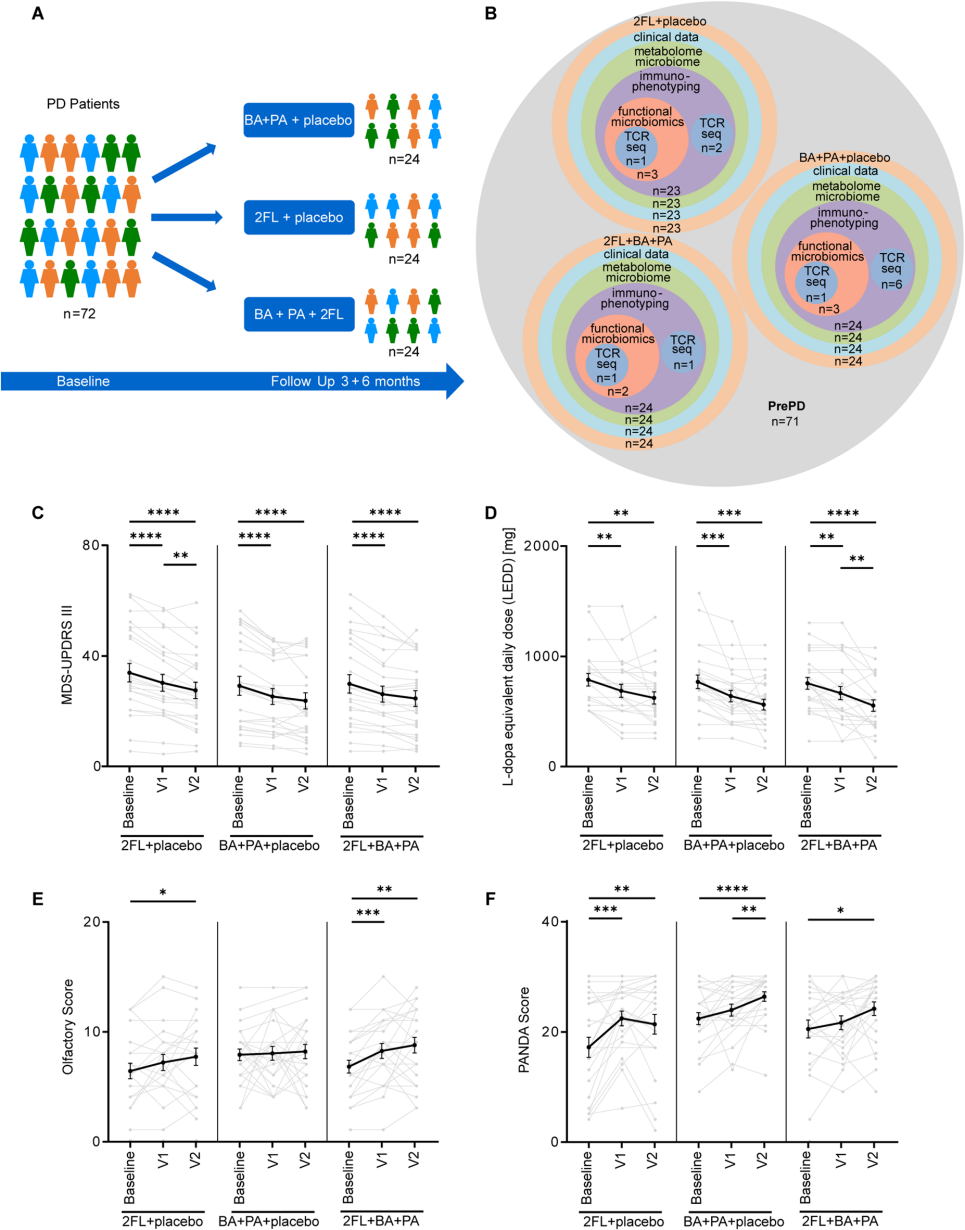
Improved clinical outcome upon supplementation. (**A, B**) Schematic representation of the randomized double-blind study. Propionic acid (PA), butyric acid (BA), 2′-fucosyllactose (2FL). Number of samples analyzed from each study group. Clinical parameters determined at the first study visit (Baseline) and at the three (V1) and six (V2) month follow-ups. (**C**) MDS-UPDRS III, (**D**) LEDD, (**E**) olfactory score and (**F**) PANDA. Data plotted as before-after for each patient (gray) and mean (black) ± SEM (BA + PA *n* = 24, 2FL *n* = 24, BA + PA + 2FL *n* = 24). Tested by 2-way ANOVA (C, D) or mixed effects analysis (**E**, **F**). **** *p* < 0.0001, *** *p* < 0.001, ** *p* < 0.01, *p < 0.05.

A total of 72 patients were included between November 2019 and August 2020 and randomly assigned to receive 2FL + placebo, PA + BA + placebo or a combination of 2FL + PA + BA. Study visits with detailed neurological examination and sample collection were performed at the beginning of the study (baseline) and after 3 (Visit1, V1) and 6 months (Visit2, V2) (Fig. 1A, B and Supplementary Fig. S1). Study groups were comparable in terms of baseline variables (Supplementary Table S3). Supplementation was generally well tolerated (Supplementary Table S4). One patient in the 2FL group discontinued treatment due to a mild nontreatment-associated adverse event, hence no follow up data are available.

To determine whether the intervention affected clinical parameters, we assessed extrapyramidal motor function using the MDS-UPDRS III^42^ and LEDD^43^, olfactory function using the Sniffin’ Sticks test^44^ and cognitive function using the PANDA^45^. All 3 interventions showed a clinically meaningful decrease in MDS-UPDRS III scores (≥ 5 points) and reduction in LEED consistently exceeded the established clinically relevant threshold of 15% over the period of 6 months (Fig. 1, C, D and Table 1). Improvement in olfaction, however, was observed only in the 2FL and combination group 2FL + BA + PA (Fig. 1E and Table 1), while PANDA revealed an improvement in cognitive functions in all groups (Fig. 1F and Table 1). Only 1 of 23 patients in the 2FL and 2 of 24 patients in the 2FL + PA + BA group reported changes to their diet after 3 months (Supplementary Table S5). In sum, 6 months of supplementation with SCFA and/or 2FL improved motor function, led to a reduction in the LEDD required and positively impacted olfactory and cognitive function in patients with PD.

**Table 1:** Secondary endpoints. Changes in clinical parameters LEED, MDS-UPRDS III, PANDA and olfactory score after 3 and 6 months of supplementation. ^a^ One way ANOVA; ^b^ Kruskal–Wallis test.

Clinical endpoints	PA + BA	2FL	2FL + PA + BA	p
	(*n* = 24)	(*n* = 23)	(*n* = 24)	
*Levodopa equivalent daily dose*				
∆% 3 months	−15.7 (± 13.6)	−13.8 (± 16.4)	−12 (± 19)	0.52^b^
95% CI	−21.5 to −10	−20.9 to −6.7	−20.1 to −4	
Percentage of patients worsening	0	0	4.2	
∆% 6 months	−25 (± 20.6)	−20 (± 22)	−26.8 (± 22.2)	0.54^a^
95% CI	−33.7 to −16.3	−29.5 to −10.4	−36.1 to −17.4	
Percentage of patients worsening	4.2	13	4.2	
*MDS-UPDRS III Change*				
∆% 3 months	−12.1 (± 6.1)	−10.8 (± 6)	−11.1 (± 6.6)	0.65^b^
95% CI	−14.6 to −9.5	−13.4 to −8.2	−13.9 to −8.3	
Percentage of patients worsening	4.2	0	0	
∆% 6 months	−18.4 (± 19.4)	−18.6 (± 14.3)	−17.9 (± 15.3)	0.94^b^
95% CI	−26.6 to −10.2	−24.8 to −12.4	−24.4 to −11.5	
Percentage of patients worsening	12.5	8.7	8.3	
*PANDA*				
∆% 3 months	2 (± 15,3)	−6,44 (± 32,6)	−6,44 (± 20,7)	0.38^a^
95% CI	−4.5 to 8.4	−20.6 to 7.7	−15.2 to 2.3	
Percentage of patients worsening	37.5	60.9	54.2	
∆% 6 months	5.4 (± 15.5)	3.2 (± 48.8)	0.1 (± 19.5)	0.7^a^
95% CI	−1.1 to −12	−17.6 to 16.7	−8.19 to 8.28	
Percentage of patients worsening	29.2	54.2	41.7	
*Olfactory score*				
∆% 3 months	15.5 (± 77.2)	22.5 (± 49.7)	26.4 (± 34)	0.8^a^
95% CI	−17.1 to 48.1	0.5 to 44.6	12.1 to 40.8	
Percentage of patients worsening	45.8	22.7	12.5	
∆% 6 months	8.5 (± 39.7)	36.4 (± 67.3)	44.2 (± 73.6)	0.11^b^
95% CI	−8.3 to 25.3	6.5 to 66.2	13.2 to 75.3	
Percentage of patients worsening	33.3	22.7	16.7	

### Gut microbiome diversity is not altered following supplementation

**Fig. 2:**
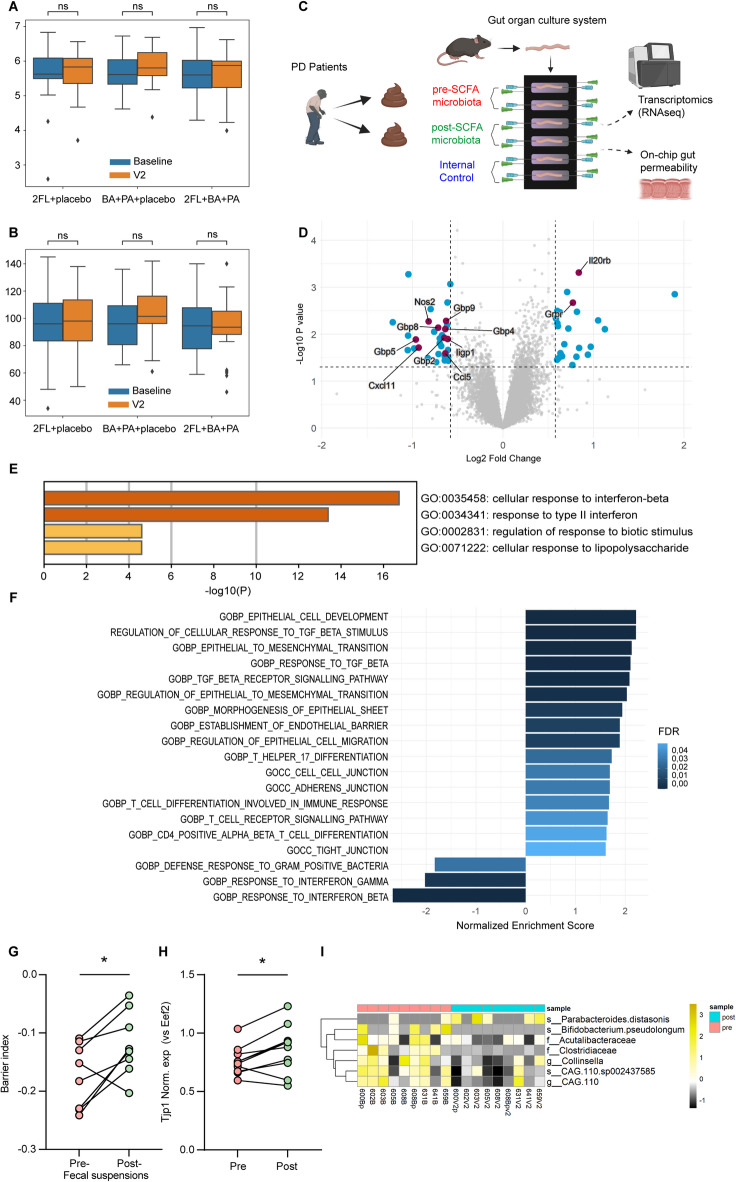
Effect of supplementation on the gut microbiome. Shotgun metagenomic sequencing of *n* = 71 stool samples (BA + PA *n* = 24, 2FL *n* = 23, BA + PA + 2FL *n* = 24). (**A**) Shannon diversity, (**B**) richness of gut microbiota at baseline and V2. (**C**) Experimental design 3D gut organ culture system. (**D**) Changes in gene expression comparing colon organ cultures infused with V2 versus baseline microbiota (*n* = 8 patients, all intervention groups included). Transcripts significantly up- or downregulated (blue), selected genes of interest based on pathway enrichment analysis (red). (**E**) GO analysis of transcripts enriched in gut cultures infused with baseline microbiota (compared with V2). (**F**) GSEA identified pathways significantly activated or inhibited following gut stimulation with post- versus preintervention microbiota. (**G**) Normalized TEER values for CaCo-2 cells cocultured with sterile fecal suspensions for 12 h (*n* = 16 in experimental triplicate, pairwise comparison per patient); representative of four independent experiments. (**H**) Tjp1 expression in CaCo-2 cells cocultured for 6 h with sterile fecal suspensions (*n* = 18 in experimental triplicate, pairwise comparison per patient); data acquired from two independent experiments. (**I**) Epithelial-adhesive microbes identified using 16S sequencing of CaCo-2 cells cocultured with fecal samples (statistically significant taxa, paired T test, *p* < 0.05). Normalized relative bacterial abundance for all taxa identified per sample. Scale-bar represent raw-normalized Z-score per taxa.

To assess whether supplementation with SCFA and/or 2FL altered microbiome diversity, we collected stool samples from all participants before and 6 months after intervention and performed shotgun metagenomic sequencing. Supplementation did not change microbiome diversity or richness in any of the study groups (Fig. 2, A and B). In addition, dimensionality reduction analysis, including the linear technique PCA and the nonlinear techniques t-SNE and UMAP, did not reveal any visual clues for differences. The concentrations of BA and PA in fecal samples were not elevated (Supplementary Fig. S2A). Similarly, the concentrations of other SCFAs remained unchanged (Supplementary Fig. S2B). Serum concentrations of BA and PA slightly increased upon supplementation (Supplementary Fig. S2C). Thus, consistent with our previous study^15^, SCFA and/or 2FL supplementation did not result in measurable alterations to gut microbiome composition.

### The microbiota of people with PD elicits distinct colonic transcriptional responses after supplementation

To determine whether intervention-induced, functional modifications to luminal content in PD patients affect colonic gene expression, we took advantage of the 3D gut organ culture system (Fig. 2C). In addition to analyzing intestinal responses to specific microbial strains^27,46^, we have recently demonstrated that this system is ideal for dissecting gut responses to human-derived, whole microbiota communities^15,47^. Colon organ cultures were infused with microbiota samples collected from PD patients at baseline and 6 months postintervention (n = 8, all intervention groups included) or with sterile medium as an internal control. Early transcriptional responses were determined by bulk RNA sequencing of colon tissues. Differential gene expression analysis comparing colonic transcriptional responses to post- versus preintervention microbiota revealed relatively mild effects of postintervention microbiota, with 54 differentially expressed genes (DEGs) (*p* value ≤ 0.05 and fold change ≥ 1.5) (Fig. 2D and Additional file 3: Data file S1). However, gene ontology (GO) analysis of DEGs indicated that the postintervention microbiota significantly inhibited pathways related to the response to IFN-β as well as responses to biotic stimulation and lipopolysaccharide (Fig. 2E). In agreement with these findings, unbiased GSEA revealed that compared to the preintervention microbiota, luminal introduction of the postintervention microbiota significantly inhibited the IFN-β and IFN-γ pathways and responses to gram-positive bacteria (Fig. 2F and Additional file 4: Data file S2). In contrast, the postintervention microbiota induced TGF-β signaling pathways as well as pathways related to epithelial adhesion, barrier functions and T-cell signaling and differentiation. Thus, SCFA and/or 2FL supplementation reduces the functional inflammatory impact of the microbiome/metabolome of PD patients.

### SCFA and/or 2FL supplementation improves gut barrier functions in vitro

As colonic responses to postintervention microbiota were enriched in pathways related to epithelial adhesion and barrier functions, and since PD is associated with leaky gut syndrome^7^, we investigated whether SCFA and/or 2FL supplementation impacts epithelial barrier integrity. TEER measurements indicated that postintervention fecal suspensions significantly ameliorated barrier disruption induced by preintervention suspensions (Fig. 2G). In agreement with these findings, we detected a significant increase in the expression of the *Tjp1* gene, which encodes the tight junction adaptor protein ZO-1 (Fig. 2H). As disruptions to barrier functions may result from close bacterial associations with the intestinal epithelium, we next investigated whether SCFA and/or 2FL supplementation might affect microbiota-epithelium associations. We found significant changes to epithelial-adhesive microbial communities postintervention (compared with preintervention; beta-diversity based on unweighted UNIFRAC, q = 0.05) (Fig. 2I and Additional file 5: Data file S3). Specifically, we detected increased levels of epithelial associated *Parabacteroides distasonis*, a human symbiont shown to strengthen the epithelial barrier and to ameliorate inflammatory and autoimmune responses^48,49^. In contrast, we detected significantly decreased levels of epithelial-associated microbes following intervention, such as decreased levels of *Collinsella*, a bacterial genus previously shown to be increased in PD patients compared with healthy controls^50,51^. Interestingly, *Collinsella* is associated with proinflammatory responses in mice and humans^52,53^ and was shown to decrease ZO-1 tight junction protein expression and to increase gut permeability^54^. Thus, postintervention microbiota improve epithelial barrier functions in vitro, potentially due to a reduced load of epithelial-adhesive microbes.

### SCFA/2FL supplementation modulates immune cell subsets

**Fig. 3:**
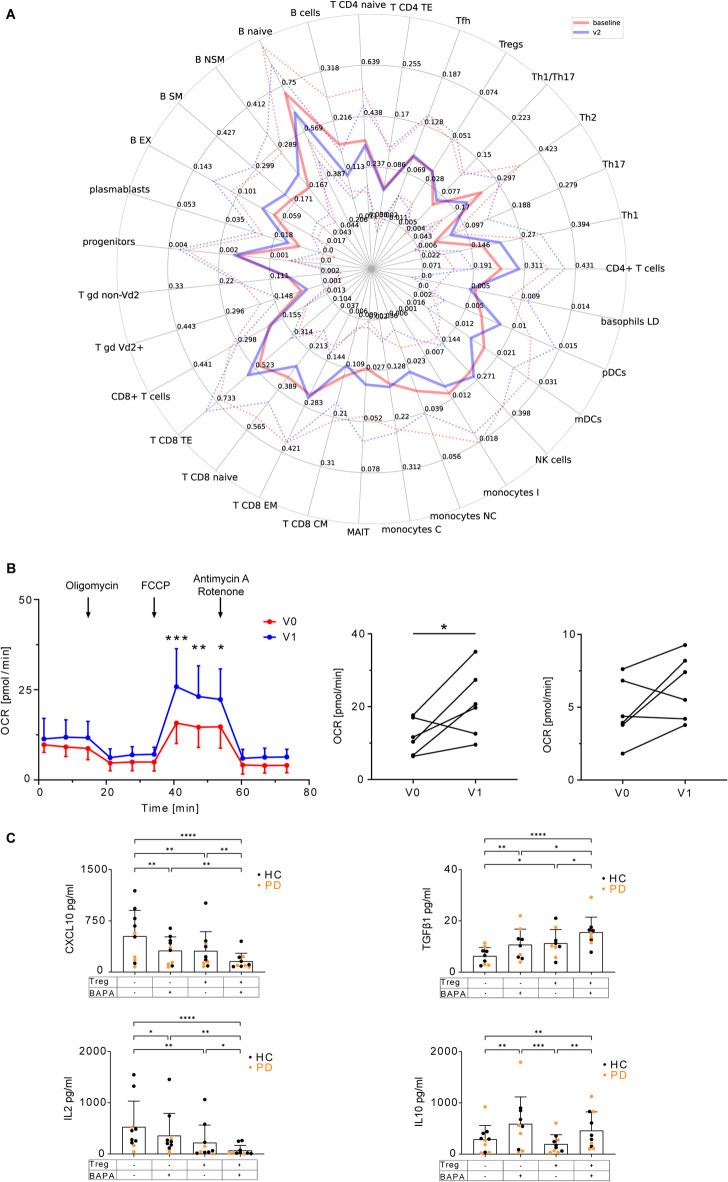
Supplementation impacts on immune cells. (**A**) Frequencies (% cells / 100%) of immune subsets at baseline (red) and 6 months (V2, blue). Mean (solid line) ± SD (dashed lines), all interventions pooled ((BA + PA *n* = 24, 2FL *n* = 23, BA + PA + 2FL *n* = 24)). T CD4 = CD4^+^ T cells, TE = terminal effector, Tfh = CD4^+^ follicular helper cells, Tregs = CD4^+^ regulatory T cells, Th = CD4^+^ T-helper cells, LD = low density, p/mDC = plasmacytoid/myeloid dendritic cells, I = intermediate, NC = non classic, C = classic, MAIT = mucosal associated invariant T cells, T CD8 = CD8^+^ T cells, CM = central memory, EM = effector memory, T gd = gamma delta T cells, progenitors = CD34^+^ progenitor cells, B EX = exhausted B cells, B SM = switched memory B cells, B NSM = non-switched memory B cells, B naïve = naïve B cells. (**B**) Mitochondrial respiration; validation cohort at baseline (V0) and after 14 days of supplementation (V1, *n* = 3 2FL or BA + PA). Left: Oxygen-consumption rate (OCR) over time, middle: Maximal and right: Basal respiration, mean ± SD, significance determined by T test. (**C**) Treg/PBMC coculture assay of *n* = 5 healthy controls (HC) or PD patients, in vitro addition of BA + PA. Data presented as mean ± SD, significance determined by Friedman multiple comparisons test.

SCFAs have been shown to exert immunomodulatory effects^15,55^; therefore, we next focused on the impact of supplementation on peripheral immune cells. We performed in-depth characterization from cryopreserved PBMCs at baseline and after 6 months. Details regarding the staining panels, subsets and gating strategies are provided in Supplementary Tables S1, S2 and Supplementary Figs. S3, S4. We detected changes in cell proportions across almost all subsets analyzed, e.g., B cells, myeloid DCs (mDC), nonclassic monocytes and Th2-T-helper cells were reduced, while the levels of plasmacytoid DCs (pDC), total CD4 and CD8 terminal effector cells increased after supplementation (Fig. 3A). Thus, supplementation with 2FL and/or SCFA induces multifaceted alterations in the composition of the peripheral immune compartment.

### SCFA/2FL supplementation modulates mitochondrial function

Since we have previously shown that SCFA improves mitochondrial respiration and restores the suppressive function of regulatory T cells (Treg) in patients with multiple sclerosis (MS)^15^, we recruited a validation cohort with *n* = 4 PD patients receiving either 2FL or BA + PA. Mitochondrial respiration was evaluated in vitro for isolated PBMCs. Maximal respiration was significantly elevated after 14 days of supplementation (Fig. 3B), suggesting that the intervention positively affected the mitochondrial function of PBMCs. In addition, we isolated Tregs from the validation cohort and performed in vitro coculture assays. We did not detect any changes in Treg suppressive capacity (Supplementary Fig. S5A), but sorted Tregs showed an increase in the expression of the mitochondrial genes *Crot* and *Xpa* after 14 days of supplementation with BA + PA (Supplementary Fig. S5B, C). We further assessed the impact of BA + PA on the functionality of Tregs by coculture experiments with and without the addition of BA + PA in vitro. Secretion of pro-inflammatory CXCL10 and IL2 was significantly decreased, while the levels of anti-inflammatory TGFβ and IL10 increased (Fig. 3C). In summary, SCFA supplementation improves mitochondrial respiration in immunocytes, inhibits the release of proinflammatory mediators and increases the secretion of anti-inflammatory mediators from immune cells.

### Clinical response is associated with distinct ex vivo colonic responses

**Fig. 4:**
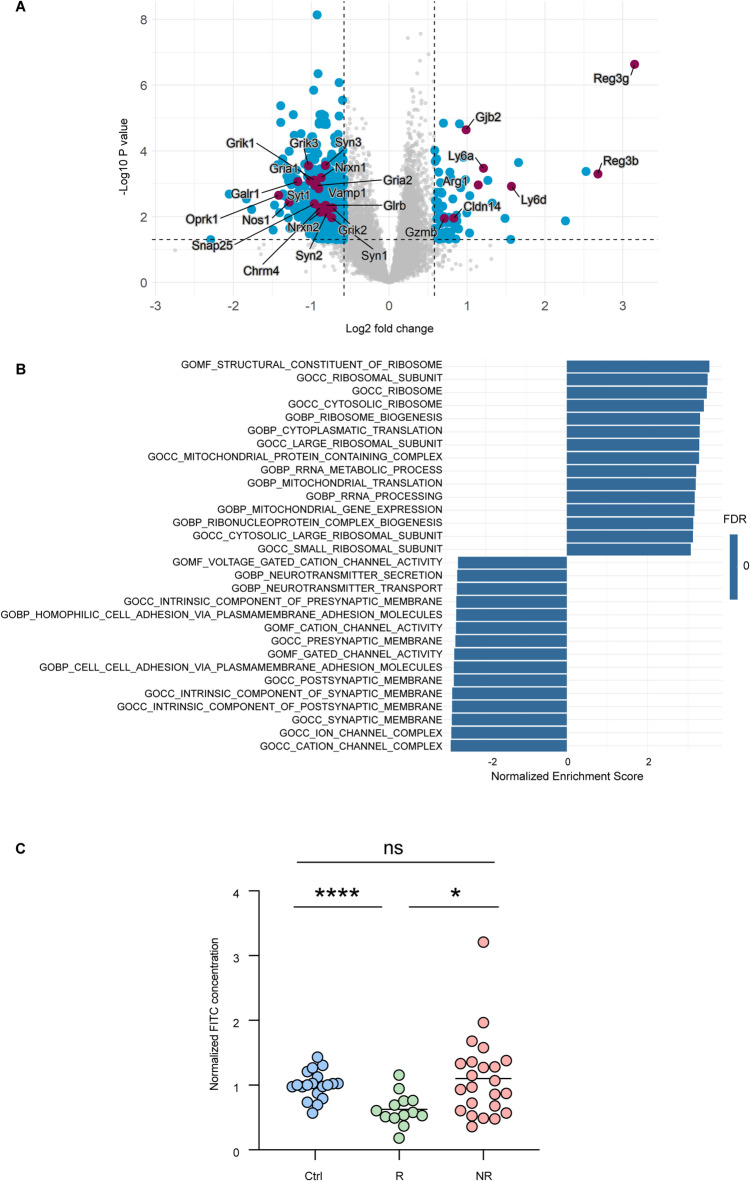
Supplementation differentially affects ex vivo colonic responses in responding patients. (**A**) Changes in gene expression after comparing colon organ cultures infused with microbiota from responding (R) and nonresponding patients (NR) (based on MDS-UPDRS III), postintervention (V2) and preintervention (*n* = 16 samples, including all intervention groups). Transcripts significantly up- or downregulated in response to R vs. NR microbiota (blue; fold change ≥ 1.5, FDR p-adjusted ≤ 0.1, Benjamini‒Hochberg), selected genes of interest (red) based on pathway enrichment analysis. (**B**) Top 15 pathways significantly activated or inhibited following gut stimulation with microbiota from responding and nonresponding patients identified by GSEA. (**C**) Ex vivo gut permeability assay. Normalized extraintestinal medium fluorescence of gut cultures infused with N or NR microbiota (normalized to sterile medium control). Statistical significance determined by unpaired T test. **** *p* < 0.0001, *p < 0.05.

While 6 months of supplementation improved motor function, this was not observed in all patients. In every intervention group, there were few patients with unchanged or increasing MDS-UPDRS III scores over time (2FL + placebo: 4/23, BA + PA + placebo: 3/24, 2FL + BA + PA: 4/24) (Supplementary Fig. S5D). To gain insight into the mechanisms underlying successful SCFA intervention, we combined all 3 intervention groups and stratified them into responders (R: MDS-UPDRS III V2 < MDS-UPDRS III baseline) and nonresponders (NR: MDS-UPDRS III V2 ≥ MDS-UPDRS III baseline). To investigate whether microbiota associated with a positive response to supplementation elicits distinct patterns of colonic gene expression, we reanalyzed the data obtained using the 3D gut organ culture system (Fig. 2C), this time comparing colonic responses to microbiota from the R and NR groups (pre- and postintervention, *n* = 16 samples). We detected broad transcriptional changes, with 42 upregulated and 451 downregulated genes (fold change ≥ 1.5, FDR p-adjusted ≤ 0.1, Benjamini‒Hochberg) (Fig. 4A and Additional file 6: Data file S4). Interestingly, the expression of genes related to mucosal barrier defense, including the antimicrobial peptides *Reg3g* and *Reg3b,* the gap junction protein *Gjb2*, and the macrophage and innate lymphoid cell marker *Arg1,* was potently induced by the R microbiota (Fig. 4A). In contrast, the NR microbiota induced the expression of numerous transcripts involved in neuronal functions, including the synaptic vesicle-associated genes *Snap25* and *Vamp1*, the glutamate receptors *Grik1*, *Grik2*, *Grik3*, *Gria1* and *Gria2*, and the muscarinic cholinergic receptor *Chrm4* (Fig. 4A). In agreement with these findings, GO and unbiased GSEA indicated that the R microbiota increased the activation of pathways related to mitochondrial functions and metabolism, while transcripts with expression that was induced by the NR microbiota were highly enriched in neuronal pathways, including neurotransmitter transport and secretion and ion-channel complexes (Fig. 4B, Supplementary Fig. S5E and Additional file 7: Data file S5).

The increased expression of genes related to barrier defense by R microbiota may be associated with improved gut permeability. We assessed this further using an ex vivo intestinal permeability assay (X-IPA) that we have recently developed^33^. Briefly, gut organ cultures were infused with R and NR microbiota in addition to FITC-dextran (4 kDa). Migration of luminal FITC-dextran to the extraintestinal culture medium depends on epithelial barrier integrity and serves as an indicator of gut permeability. Consistent with the transcriptional data, luminal infusion of microbiota from responding PD patients led to less luminal FITC-dextran leakiness and better gut permeability than the use of NR microbiota or internal controls (tissue infused with sterile medium) (Fig. 4C). Thus, luminal introduction of microbiota from responding patients improves gut barrier functions and remodels metabolic and neuronal gene expression, unlike NR microbiota.

### Distinct changes in immune cell subsets in responding patients

**Fig. 5:**
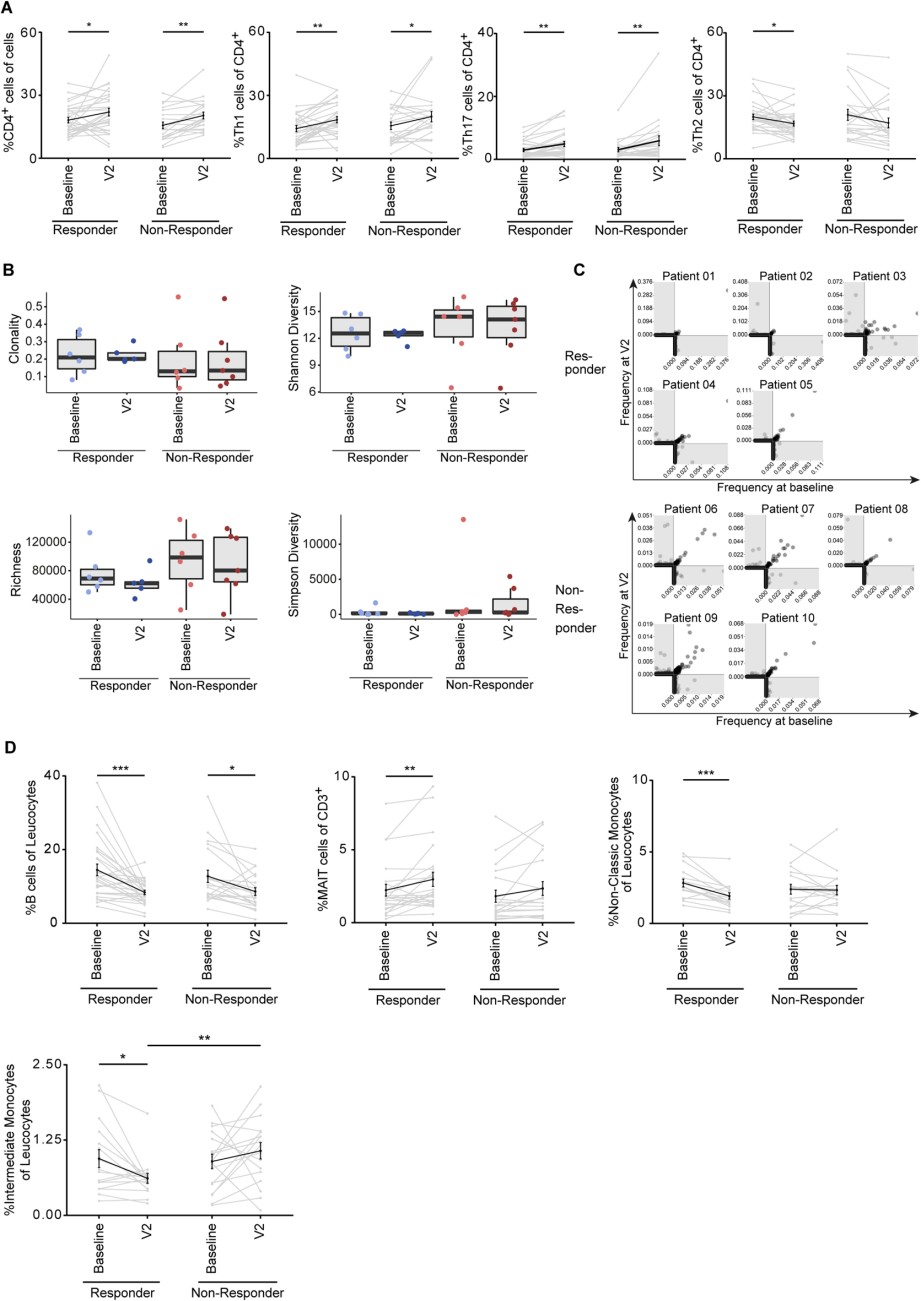
Supplementation differentially affects immune cell subsets in responding patients. (**A**) Proportions of CD4-, Th1-, Th17- and Th2 T cells among PBMCs, depicted as median split MDS-UPDRS III responder/nonresponder, matched baseline and V2, mean ± SEM. Significance was determined by paired T test. (pooled analysis, Responder *n* = 36, non-Responder *n* = 35;) (**B**) TCR- repertoire metrics: Clonality, richness and diversity indices. Each point represents one repertoire, Tukey boxplots (*n* = 8 responder, *n* = 8 nonresponder at baseline and after 6 months). (**C**) Overlap scatterplots of paired patient samples (*n* = 8 responder, *n* = 8 nonresponder at baseline and after 6 months). Each point represents a single TCR-β clone plotted according to its fraction at baseline (x-axis) and V2 (y-axis). White quadrant: Clones with identical CDR3 amino acid sequences present at both time points, gray area: Clones present at only one time point. (**D**) Proportions of B cells, MAIT, nonclassic and intermediate monocyte populations in PBMCs depicted as median split MDS-UPDRS III responder/nonresponder, matched baseline and V2, mean ± SEM (pooled analysis, Responder *n* = 36, non-Responder *n* = 35;). Significance was determined by paired T test. *** *p* < 0.001, ** *p* < 0.01, *p < 0.05.

We stratified the data from our in-depth immune cell study into R and NR (median split on % change in MDS-UPDS III) and detected significant increases of CD4 T cells after 6 months of intervention (Fig. 5A). Supplementation differentially affected subsets of CD4 T cells, with significantly increased Th1- and Th17 cells in the R and NR groups, while the levels of Th2 cells were significantly reduced only in responding patients (Fig. 5A). The comparison of CD4 terminal effector and naïve cells did not show any significant differences (Supplementary Fig. S6). TCR-β sequencing revealed no significant differences in the clonality, diversity or richness of the T-cell repertoire (Fig. 5B). There was a trend toward less rich and more clonal repertoires only in responders, which was even more apparent after supplementation. Consistent with this finding, responding patients had more hyperexpanded T-cell clones (Supplementary Fig. S7A). We did not detect skewing of TRBV gene usage (Supplementary Fig. S7B). Interestingly, we found fewer shared clones in responders at baseline and follow-up, especially within the hyperexpanded pool, indicating the selection of novel T-cell clones (Fig. 5C). However, comparison with known sequences, including α-synuclein, (VDJdb: https://vdjdb.cdr3.net/)^40^ did not reveal the expansion of T-cell clones with published epitope specificities (Supplementary Fig. S7C, D).

The levels of B cells were also significantly reduced both in the R and NR groups (Fig. 5D), while changes in naïve and nonswitched memory B cells were detected only in responders, whereas nonresponders showed a significant increase in the level of plasmablasts upon supplementation (Supplementary Fig. S6). The percentages of CD4 Tfh and Treg cells, leukocyte progenitors, CD8 T cells, subsets of γδ-T cells, NK cells, mDCs, pDCs, classic monocytes and basophils were not significantly altered and did not differ between responders and nonresponders (Supplementary Fig. S6). Remarkably, the percentages of MAIT and nonclassic and intermediate monocyte subsets only significantly changed in responders (Fig. 5D). In summary, supplementation with 2FL and/or SCFA induces a multitude of changes in the composition of immune cell subsets in the blood regardless of treatment response, while increased numbers of MAIT cells and decreased numbers of nonclassic and intermediate monocytes seem to correlate exclusively with intervention-induced clinical improvement.

### Multiobjective analysis reveals parameters associated with best response to intervention

**Fig. 6:**
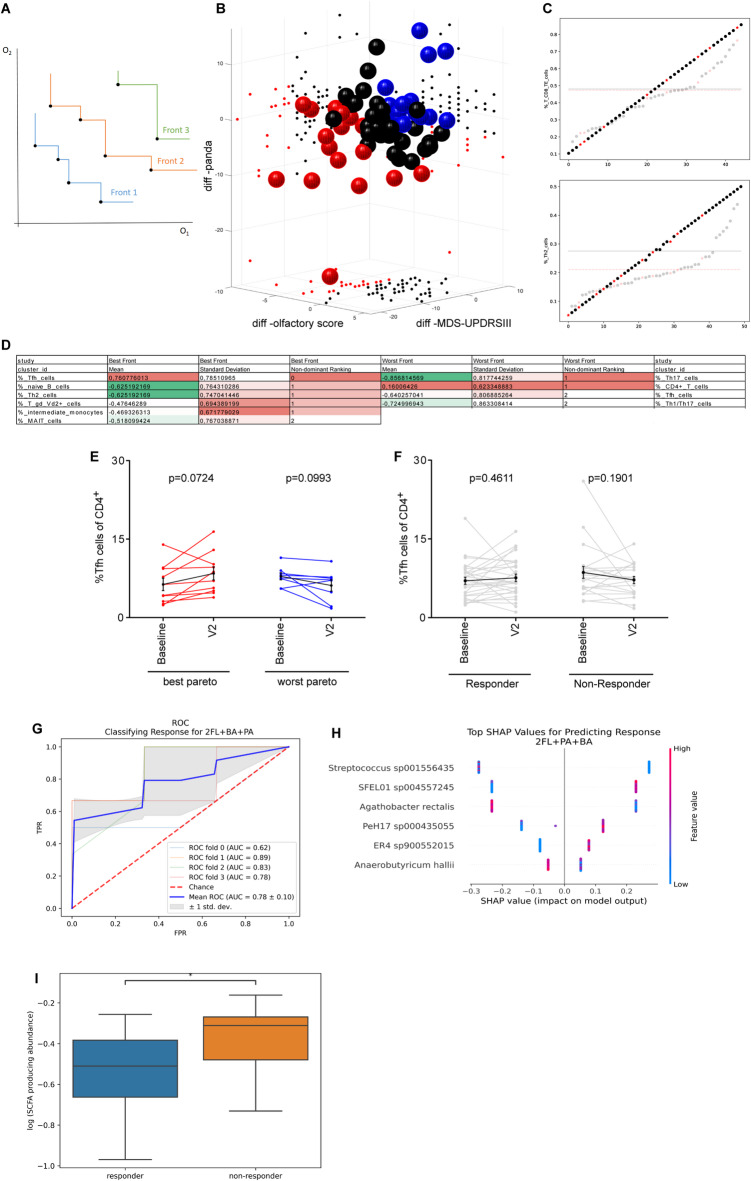
Multiobjective analysis and prediction modeling reveal parameters associated with the best/worst response to intervention. (**A**) Example of nondominated sorting. (**B**) Nondominated sorting based on olfactory score, MDS-UPDRS III and PANDA (V2 – baseline). The clusters of patients with the best (red) and worst (blue) 20% performances. (**C**) Examples of an uncorrelated (upper panel) and a correlated parameter (lower panel). Solid points depict ranking (not to scale), and transparent points the raw values, data points in the best cluster (red). Mean of all rankings (solid black line) and mean only in the best cluster (dashed red line). (**D**) Nondominated ranking of physiological parameters associated with the best/worst response. (**E**) Proportions of CD4 Tfh cells from patients within the best and worst clusters, matched baseline and V2, black line mean ± SEM. (**F**) Proportions of CD4 Tfh cells in all patients, median split MDS-UPDRS III responder/nonresponder, matched baseline and V2, black line mean ± SEM (pooled analysis, Responder *n* = 36, non-Responder *n* = 35;). Significance was determined by paired T test. (**G**) Prediction model for distinguishing R from NR: ROC curve of a prediction model based solely on microbiome baseline features (blue). (**H**) SHAP analysis of the model. (**I**) Abundances of SCFA-producing bacteria before supplementation with 2FL + BA + PA, *p < 0.05.

We initially defined responders and nonresponders by a reduction in MDS-UPDRS III after 6 months of supplementation. However, this method did not consider patients showing improvement in olfactory and/or cognitive function or improvement in one score but worsening in another score. Therefore, we devised an MOA to incorporate the various clinical measurements and thus more accurately determine the response to intervention. Multiobjective problems usually contain a subset of data points that are not dominated by others in the dataset and are located on a front surface if mapped to the objective space. By performing the domination criterion on all the data points, they can be sorted into fronts, where the first and last fronts mark the best and worst in terms of the objectives (Fig. 6A). We performed nondominated sorting on the clinical parameter difference (V2 – baseline) with three metrics (objectives): Olfactory score, MDS-UPDRS III and PANDA. After obtaining the front numbers, we selected the 20% of patients who had the lowest and highest front numbers and sorted them into two clusters. These clusters included patients with the best/worst response to intervention based on the three studied clinical metrics (Fig. 6B). One major feature in our dataset concerns the different scales and distributions of the parameters, which makes it difficult to use existing statistical testing methodologies^56^. Therefore, we performed binary correlation-based feature selection to identify the physiological parameters that have the strongest correlation with the best/worst response to intervention. We calculated the ratio ln(V2/baseline) for every determined physiological parameter and converted entries into rankings. Then, we normalized the rankings (mean of 0 and standard deviation of 1). Within each cluster, we computed the mean and standard deviation of the parameters. In this case, the mean describes the level of deviation of a particular parameter within a cluster from the rest of the population, while the standard deviation describes the level of concentration compared to the rest of the population. Using these two values, we identified the parameters with high levels of deviation that also had low levels of variation within a cluster (Fig. 6C). This approach enabled the identification of parameters associated with the best or worst combined clinical response (Fig. 6D). While some of the immune parameters already showed significant correlations when response was solely defined by changes in MDS-UPDRS III, this novel approach of MOA revealed additional immune cell subsets associated with response to intervention. We identified CD4 Tfh cells as the parameter with the highest correlation to clinical response (Fig. 6E). This association was not evident when immune cell data were stratified by median split on MDS-UPDRS III (Fig. 6F). Thus, MOA is a promising novel approach to identify parameters associated with response to intervention in complex datasets with several conflicting metrics describing clinical outcome and a highly diverse repertoire of metrics recorded as potential correlates.

#### PD patients’ microbiome before 2FL + BA + PA supplementation is associated with response

To assess whether the response to supplementation was affected by the patients’ microbiome, we used baseline microbiome abundances, as they may hold a potential to predict the impact of intervention. We devised a prediction model utilizing an XGBoost classifier. The model solely uses baseline microbiome data as its inputs and outputs a prediction of the treatment response. Response was treated as a binary variable (median split on % change in MDS-UPDS III). The model performance was highly dependent on the supplementation type. The model based on the microbiome of patients who received only 2FL or BA + PA had no predictive capability. However, the model predicted response with an area under the receiver operating characteristic curve (AUC) of 0.77 ± 0.07 (Fig. 6G) when it was established based on the baseline microbiome of participants who received 2FL + BA + PA. We conducted SHapley Additive exPlanations (SHAP) analysis (Fig. 6H) to understand which species had the greatest impact on the model’s prediction. Our analysis revealed that *Streptococcus* sp001556435 and *Agathobacter rectalis* contributed to the prediction of nonresponders, whereas SFEL01 sp004557245 had a significant impact on the prediction of responders. *Agathobacter rectalis* is a SCFA-producing bacterium. Interestingly, our study also revealed that the total abundance of SCFA-producing bacteria was significantly different between responders and nonresponders who were treated with 2FL + BA + PA; responders had a lower abundance of SCFA-producing bacteria (Fig. 6I).

## Discussion

Here, we report that supplementation with SCFA improves clinical outcome in PD patients in a randomized double-blind 6-month prospective study with 72 patients. To our knowledge, this is the first exploratory study of SCFAs in PD patients showing improvement in motor and nonmotor functions beyond the level provided by standard medication. This was observed in all intervention groups (2FL + placebo, BA + PA + placebo, 2FL + BA + PA), likely due to shared mechanisms of action that are mediated by SCFAs and prebiotics such as 2FL that are converted to SCFAs in the intestine^57^. Collectively, SCFA supplementation led to improvements in motor, cognitive, and olfactory function, consistent with systemic effects across multiple brain regions. Motor outcomes approached or exceeded clinical relevance, with MDS-UPDRS III scores showing reductions at or beyond the minimal clinically important difference (≥ 5 points), paralleled by LEDD decreases of more than 15%^58,59^. In contrast, effects on nonmotor domains were more heterogeneous, with olfactory improvements observed primarily in the 2FL and combination groups. Amelioration of motor and nonmotor symptoms suggests a neuroregenerative element of SCFA supplementation. We have recently shown that recovery of damaged neurites was induced by PA and BA in a *disease-in-a-dish* model, mediated via the free fatty acid receptor signaling pathway, histone deacetylase inhibition and antioxidative mechanisms^60^. While we were not able to collect cerebrospinal fluid (CSF) in this study, we have previously reported increased PA concentrations in CSF after supplementation in MS patients^15^. In addition, free fatty acid receptor 3 (Ffar3) is expressed on the human brain endothelium, and its interaction protects the BBB from oxidative stress^61^. Thus, supplementation with SCFAs may also directly influence the BBB and central nervous system (CNS) to promote neuroregeneration. However, given the limited 6 month observation period, the improvements observed here should be interpreted with caution, as they are more likely to reflect symptomatic relief than long-term disease modification. To clarify whether such effects extend beyond symptomatic benefits, future research will require longer follow-up periods and the integration of biomarker analyses.

Supplementation did not alter microbial composition. Even though microbial dysbiosis has been reported in PD patients, our results suggest that amelioration of disease symptoms by SCFA and/or 2FL supplementation does not require profound changes in gut microbe populations, but rather changes in the microbiome function.

It is well established that the majority of SCFAs produced by the microbiota or administered orally are rapidly metabolized by colonocytes and the liver, which explains why serum levels generally underestimate local concentrations in the colon^62^. Consequently, systemic elevations after oral substitution are only transient, returning to baseline within a few hours, and thus not expected to drive sustained clinical effects^63^. In line with this, we observed only marginal increases in circulating SCFAs. This supports the notion that the biological relevance of supplementation is more likely mediated by local colonic mechanisms such as barrier stabilization and immune regulation rather than by persistent changes in serum concentrations. Notably, previous studies demonstrated that even lower dosages (2 × 500 mg PA/day) exerted clinically relevant effects, further highlighting the critical role of local rather than systemic SCFA activity^15^.

The SCFA concentration in fecal samples also remained unaffected by supplementation, suggesting that excess SCFA is not excreted via stool. Over 95% of SCFAs produced in the colon are absorbed by the gut mucosa in healthy individuals^64^ or transported via the portal vein to the liver, preventing escape into the systemic circulation^65^. Since SCFAs are not passed in the stool and serum levels only marginally increase, SCFAs are most likely swiftly metabolized. This supports our interpretation that systemic or local effects of SCFAs may occur without measurable changes in fecal concentrations, and that functional microbial alterations below the resolution of global diversity metrics may still underlie the clinical improvement observed.

Leaky gut syndrome in addition to increased levels of proinflammatory luminal factors has been postulated to be involved in PD pathogenesis^7^. Here, we found that luminal contents collected post-SCFA intervention reduced inflammatory gene expression and reinforced epithelial barrier functions in cells and organ cultures compared with preintervention suspensions. However, it must be noted that species-specific differences in gene expression may limit the direct transferability of our findings to human PD and further verification e.g. in human colon organoid cultures is required. Potentially, these effects could be mediated by direct sensing of luminal SCFAs by intestinal epithelial cells, i.e. via free fatty acid receptors, resulting in enhanced barrier defense^66–68^. Person-to-person heterogeneity in intestinal responses to SCFAs has an impact on clinical outcomes. Indeed, we have demonstrated that luminal infusion of microbiota from responding patients into gut organ cultures reduced gut ‘leakiness’ and induced barrier-related gene expression, unlike microbiota from nonresponders. In this context, we detected regenerating islet-derived protein type 3 (Reg3) family members *Reg3b* and *Reg3g* among the highly upregulated genes with expression that was induced by microbiota from responders. Reg3 is an antimicrobial peptide produced in the gut mucosa that plays an important role in maintaining gut homeostasis and possesses bactericidal activity^69^. It restricts the activity of the mucosa-associated microbiota and prevents translocation of commensal organisms after tissue injury, and a lack of Reg3b has been associated with microbial dysbalance^70,71^. Importantly, Reg3 also promotes tissue regeneration after injury^72^. Recently, PA, but not BA, has been shown to induce Reg3 expression in intestinal organoids via Ffar2, implicating that the Reg3-PA axis is an important mediator of gut epithelial regeneration in colitis^73^. Thus, SCFA supplementation may improve barrier integrity and contribute to intestinal regeneration by stimulating the secretion of antimicrobial Reg3 family members and inhibiting gut inflammation, which promotes disease progression.

In addition to a direct neuroregenerative effect of PA and BA, an anti-inflammatory mechanism may also account for the improvement of both motor and nonmotor symptoms. Growing evidence supports the notion that the immune system plays a critical role in the pathogenesis of idiopathic PD^74–76^. The anti-inflammatory effect of SCFAs, with subsequent improvement in neurodegenerative conditions, is well established^15^. In PD, the supplementation led to a significant increase in MAIT cells only in responding patients. At barrier surfaces, MAIT cells mediate the crosstalk between the host, the metabolome and the microbiome^77^. Importantly, reduced numbers of MAIT cells in peripheral blood have been reported in patients with several autoimmune diseases^78^ and MAIT cells play a regulatory role in neuroinflammation through suppression of pathogenic Th1 cells^79^. Recently, a TCR-mediated protective effect of MAIT cells accumulated in the inflamed CNS via amphiregulin has been shown in a mouse model of MS^80^. This suggests MAIT cells might also partake in dampening neuroinflammation in PD. Furthermore, MAIT cells secrete IL-17 and IL-22 to strengthen epithelial/mucosal barriers and could thus potentially also stabilize the BBB. This crucial barrier function could prevent the influx of pathogenic agents and pro-inflammatory molecules into the CNS, thus mitigating the inflammatory processes implicated in PD.

We observed a significant decrease in non-classic and intermediate monocytes only in responding patients after supplementation. A shift toward proinflammatory states in monocyte populations with a decrease in the levels of classic subsets and an increase in the levels of intermediate subsets with increased HLA-DR expression has been shown in PD^81^, and a large study on expression quantitative trait loci (eQTL) revealed the overrepresentation of PD-related genes in monocyte populations^82^. Several genome-wide association studies have identified single-nucleotide polymorphisms in HLA genes associated with PD. Increased baseline expression of MHCII and greater inducibility of MHCII expression on B cells and monocytes have been reported, thus implicating the role of increased MHCII-dependent antigen presentation in promoting PD^83,84^. Decreased absolute counts of Tfh cells have also recently been reported in PD patients^85^, in addition to decreased numbers of circulating anti-inflammatory B-cell subsets, thus implicating defective B-cell regulation as another contributing factor in PD. Interestingly, our MOA revealed increased frequencies of Tfh cells as the parameter with the highest correlation to clinical response. In summary, we propose that supplementation with SCFAs acts in concert on innate-like T cells, monocytes and B cells to promote a reduced inflammatory immune response in PD patients. This shift to a less inflammatory profile could also explain the broad improvement in Parkinson’s symptoms.

Our prediction model for response suggests that the patient microbiome can predict the efficacy of 2FL and BA + PA supplementation. Specifically, the composition of the SCFA-producing gut microbiota affected the response, with responders having less SCFA-producing gut microbes, which could help explain why supplementation with SCFA induces a greater response rate among them. If validated in larger studies, such a model can help evaluate the chances of a specific patient benefitting from intervention before it is started.

## Conclusions

Supplementation with SCFAs and/or 2FL in addition to standard medication significantly ameliorated motor and nonmotor symptoms in people with PD. Our data suggest a multifactorial mechanism of action that involves strengthening of the intestinal epithelial barrier, improvement of mitochondrial functions, anti-inflammatory modulation of immune cell subsets and possibly also direct neuroregeneration. In summary, SCFA supplementation may be a promising disease-modifying strategy in PD, hence, a follow-up phase III clinical trial to investigate the therapeutic potential of SCFAs in PD is warranted.

### Limitations

The sample size is insufficient to draw definitive conclusions regarding the clinical efficacy of the intervention. Therefore, further research with a larger cohort is necessary to validate these findings and establish more conclusive evidence.

## Supplementary Information

Below is the link to the electronic supplementary material.

Supplementary Material 1

Supplementary Material 2

Supplementary Material 3

Supplementary Material 4

Supplementary Material 5

Supplementary Material 6

Supplementary Material 7

Supplementary Material 8

## Data Availability

All datasets generated and/or analysed in the current study are freely available in the Gene Expression Omnibus under the GEO accession number GSE296010 and GSE296011. Any additional information required to reanalyze the data reported in this work is available from the corresponding author upon request. This paper does not report any original code or algorithms**.** The code used in this study is freely available at https://www.ci.ovgu.de/Research/Codes.html and on GitHub.

## Abbreviations

2FL: 2′-Fucosyllactose
BA: Butyric acid
BBB: Blood‒brain barrier
CNS: Central nervous system
CTRL: Control
DEG: Differentially expressed gene
FC: Fold change
FITC: Fluorescein isothiocyanate
GO: Gene ontology
GSEA: Gene set enrichment analysis
HC: Healthy control
LEED: Levodopa equivalent daily dose
MAIT: Mucosal-associated invariant T cells
MDS-UPDRS III: The movement disorder society-sponsored revision of the Unified Parkinson’s disease rating scale
MOA: Multiobjective analysis
MS: Multiple sclerosis
NR: Nonresponder
OCR: Oxygen consumption rate
PA: Propionate
PANDA: Parkinson neuropsychometric dementia assessment
PBMC: Peripheral blood mononuclear cell
PCA: Principal component analysis
PD: Parkinson’s disease
R: Responder
SCFA: Short-chain fatty acid
TCR: T-cell receptor
TEER: Transepithelial electrical resistance
Tfh: T follicular helper cells
Th: T-helper cells
Treg: Regulatory T cell
t-SNE: T-distributed stochastic neighbor embedding
UMAP: Uniform manifold approximation and projection
V1: Visit 1, follow up 3 months
V2: Visit 2, follow up 6 months
X-IPA: Ex vivo Intestinal permeability assay
