# Supplementary material for: Supplementation with short-chain fatty acids and a prebiotic improves clinical outcome in Parkinson’s disease: a randomized double-blind prospective study
Source: Sci Rep. 2025 Dec 5;16:315. doi: 10.1038/s41598-025-29692-x (PMC12770440; doi:10.1038/s41598-025-29692-x)
Supplement: Supplementary file 3 — Supplementary Material 3 [file 41598_2025_29692_MOESM3_ESM.pdf]

Surface Marker	Clone	Fluorochrome	Company	Cat-Nr.	Panel
CD3	UCHT1	FITC	BioLegend	300440	1, 2, 4
CD4	RPAT4	APC Fire 750	BioLegend	300560	1
CD25	M-A251	BV785	BioLegend	356140	1
CD127	A019D5	APC	BioLegend	351316	1
CXCR5	J25204	PE/Dazzle 594	BioLegend	356928	1
CD45RA	H100	BV605	BioLegend	304134	1,2
CCR7	G043H7	PerCP/Cy5.5	BioLegend	353220	1,2
CCR6	11A9	PE	BD	559562	1
CXCR3	G025H7	BV650	BioLegend	353730	1
CCR4	L2A1H4	BV421	BioLegend	359414	1
CD8	SK1	APC Fire 750	BioLegend	344746	2
CD161	HP3G10	PE	BioLegend	339904	2
Vδ7.2	3C10	BV785	BioLegend	351722	2
TCR γ/δ	11F2	APC	Miltenyi	130-113-500	2
Vδ2	B6	BV421	BioLegend	331428	2
CD19	H1B19	FITC	BioLegend	302206	3, 4
IgD	1A6-2	PE/Dazzle 594	BioLegend	348240	3
CD45	H130	Alexa Fluor 700	BioLegend	304024	3, 4
CD27	323	BV421	BioLegend	302824	3
CD38	HIT2	APC	BioLegend	303510	3
CD34	563	PE	BD	550761	3
CD11c	B-LY6	APC	BD	559877	4
CD14	HCD14	PE/Dazzle 594	BioLegend	325634	4
CD16	3G8	APC Fire 750	BioLegend	302060	4
CD56	HCD56	PerCP/Cy5.5	BioLegend	318322	4
CD11b	ICRF44	PE	BioLegend	301306	4
CD123	6H6	BV605	BioLegend	306026	4
HLA-DR	L243	BV785	BioLegend	307642	4

Supplementary Table S1: List of antibodies used for in-depth immunophenotyping.

Panel 1 - CD4 T cells			
**Cell type**	**Gating strategy**		
CD4+ T cells	CD3^{+} CD4^{+}		
T follicular helper (Tfh)		CXCR5^{+}	
T regulatory cells (Tregs)		CXCR5^{-} CD25^{high} CD127^{low}	
T helper 1 (Th1)		CXCR5^{-} CD127^{+} CD25$^{-/low}$ CXCR3^{+} CCR6^{-}	
T helper 1/T helper 17 (Th1/Th17)		CXCR5^{-} CD127^{+} CD25$^{-/low}$ CXCR3^{+} CCR6^{+}	
T helper 17 (Th17)		CXCR5^{-} CD127^{+} CD25$^{-/low}$ CXCR3^{-} CCR6^{+}	
T helper 2 (Th2)		CXCR5^{-} CD127^{+} CD25$^{-/low}$ CXCR3^{-} CCR6^{-} CCR4^{+}	
T CD4 terminal effector (T CD4 TE)		CXCR5^{-} CD127^{+} CD25$^{-/low}$ CXCR3^{-} CCR6^{-} CCR4^{-} CCR7^{-} CD45RA^{low}	
T CD4 Naïve		CXCR5^{-} CD127^{+} CD25$^{-/low}$ CXCR3^{-} CCR6^{-} CCR4^{-} CCR7^{+} CD45RA^{high}	
**Panel 2 - CD8, γ/δ and MAIT T cells**			
**Cell type**	**Gating strategy**		
CD8+ T cells	CD3^{+}	TCR γ/δ^{-}	CD16^{high} NOT Vα7.2^{+}
γ/δ Vd2^{+} (T gd Vd2+)		TCR γ/δ^{+}	Vd2^{+}
γ/δ Vd2^{-} (T gd non-Vd2)			Vd2^{-}
MAIT		TCR γ/δ^{-} Vd2^{-}	Vα7.2^{+} CD161^{high}
T CD8 Naïve		CD8^{+} TCR γ/δ^{-} Vd2^{-}	CCR7^{+} CD45RA^{+}
T CD8 central memory (CM)			CCR7^{+} CD45RA^{-}
T CD8 effector memory (EM)			CCR7^{-} CD45RA^{-}
T CD8 terminal effector (TE)			CCR7^{-} CD45RA^{+}
**PANEL 3 - B cells and Progenitors**			
**Cell type**	**Gating strategy**		
Progenitors	CD45^{+}	CD34^{+} CD45^{low}	
Total B cells (B cells)		CD19^{+} CD34^{-}	
Naïve B cells (B naïve)			CD27^{-} IgD^{+}
Nonswitched memory B cells (B NSM)			CD27^{+} IgD^{+}
Exhausted B cells (B EX)			CD27^{-} IgD^{-}

Switched memory B cells (B SM)			$CD27^+$ IgD^- $CD38^{low}$
Plasmablasts			$CD27^+$ IgD^- $CD38^{high}$
**Panel 4 - Monocytes, DCs, NK cells, Low-density basophils**			
**Cell type**	**Gating strategy**		
NK cells	$CD45^+$ $CD3^-$ $CD19^-$		$CD16^{+/-}$ $CD56^{+/low}$ $CD14^-$ $CD11c^-$
Classic monocytes (C)			$CD14^+$ $CD16^-$
Intermediate monocytes (I)		$CD11c^+$	$CD14^+$ $CD16^+$
Nonclassic monocytes (NC)			$CD14^{low}$ $CD16^+$
Myeloid dendritic cells (mDCs)		$CD16^-$ $CD56^-$	$HLA\text{-}DR^+$ $CD11c^+$ $CD123^{low}$
Plasmacytoid dendritic cells (pDCs)			$HLA\text{-}DR^+$ $CD11c^-$ $CD123^+$
Low-density basophils (LD)			$HLA\text{-}DR^-$ $CD123^+$ $CD11b^+$

Supplementary Table S2: Gating strategies for in-depth immuno-phenotyping of immune cell subsets.

Characteristics	PA+BA+placebo (n=24)	2FL+placebo (n=24)	2FL+PA+BA (n=24)
Female sex	13 (54.2%)	9 (37.5%)	13 (54.2%)
Age, years	62(±9)	67 (±12)	65 (±8)
Duration of disease, years	7 (±7)	6 (±6)	4 (±4)
BMI	26 (±4)	26 (±3)	25 (±3)
Subgroups			
akinetic-rigid	8 (33.3%)	9 (37.5%)	11 (45.8%)
equivalent	14 (58.3%)	12 (50%)	12 (50%)
tremordominant	2 (8.3 %)	3 (12.5%)	1 (4.17%)
Medication			
LEDD [mg]	770 (±302)	785 (±267)	756 (±261)
95% CI	642.1-897.1	672.2-897.3	646-866.5
Benserazide	24 (100%)	22 (91.7%)	23 (95.8%)
Carbidopa	4 (16.7%)	5 (20.8%)	2 (8.3%)
Piribedil	2 (8.3%)	0	2 (8.3%)
Rotigotine	2 (8.3%)	3 (12.5%)	3 (12.5%)
Ropinirole	2 (8.3%)	1 (4.17%)	2 (8.3%)
Pramipexole	4 (16.7%)	2 (8.3%)	4 (16.7%)
Rasagiline	13 (54.2%)	8 (33.3%)	14 (58.3%)
Selegiline	3 (12.5%)	9 (37.5%)	3 (12.5%)
Safinamide	2 (8.3%)	1 (4.2%)	2 (8.3%)
COMT-inhibitor	6 (25%)	8 (33.3%)	9 (37.5%)
Amantadine	2 (8.3 %)	2 (8.3 %)	1 (4.2%)
Apomorphine	0	0	0
Anticholinergics	0	1 (4.2%)	0
Clozapine	1 (4.2%)	0	2 (8.3%)
Quetiapine	1 (4.2%)	3 (12.5%)	1 (4.2%)
PD Clinical Scores			
MDS-UPDRS I	10 (±10)	17 (±12)	17 (±13)
95% CI	12.6-21	12.1-22.5	11.2-22.1
MDS-UPDRS II	9 (±6)	9 (±8)	10 (±9)

95% CI	6.8-12.1	6.3-12.7	6-13.4
MDS-UPDRS III	29 (±17)	33 (±16)	30 (±16)
95% CI	22.1-36.3	26.3-40	23-36.8
MDS-UPDRS IV	3 (± 3)	5 (±5)	3 (±3)
95% CI	1.8-4.2	2.5-6.3	1.4-4.1
Hoehn & Yahr scale	2 (±1)	2 (±1)	2 (±1)
95% CI	1.9-2.5	1.9-2.4	1.9-2.7
PANDA	25 (±4)	33 (±16)	30 (±16)
95% CI	23.7-26.7	18.9-24.8	20.8-26.1
Olfactory score	8 (±3)	7 (±3)	7 (±3)
95% CI	6.8-9	5.9-8.1	5.6-8.1

Supplementary Table 3. Demographic data and characteristics of patients prior to therapeutic intervention. Main demographics and clinical and treatment characteristics of participants at study entry. Data are presented as the mean ± SD or n (%).
