# Supplementary material for: Supplementation with short-chain fatty acids and a prebiotic improves clinical outcome in Parkinson’s disease: a randomized double-blind prospective study
Source: Sci Rep. 2025 Dec 5;16:315. doi: 10.1038/s41598-025-29692-x (PMC12770440; doi:10.1038/s41598-025-29692-x)
Supplement: Supplementary file 7 — Supplementary Material 7 [file 41598_2025_29692_MOESM7_ESM.pdf]

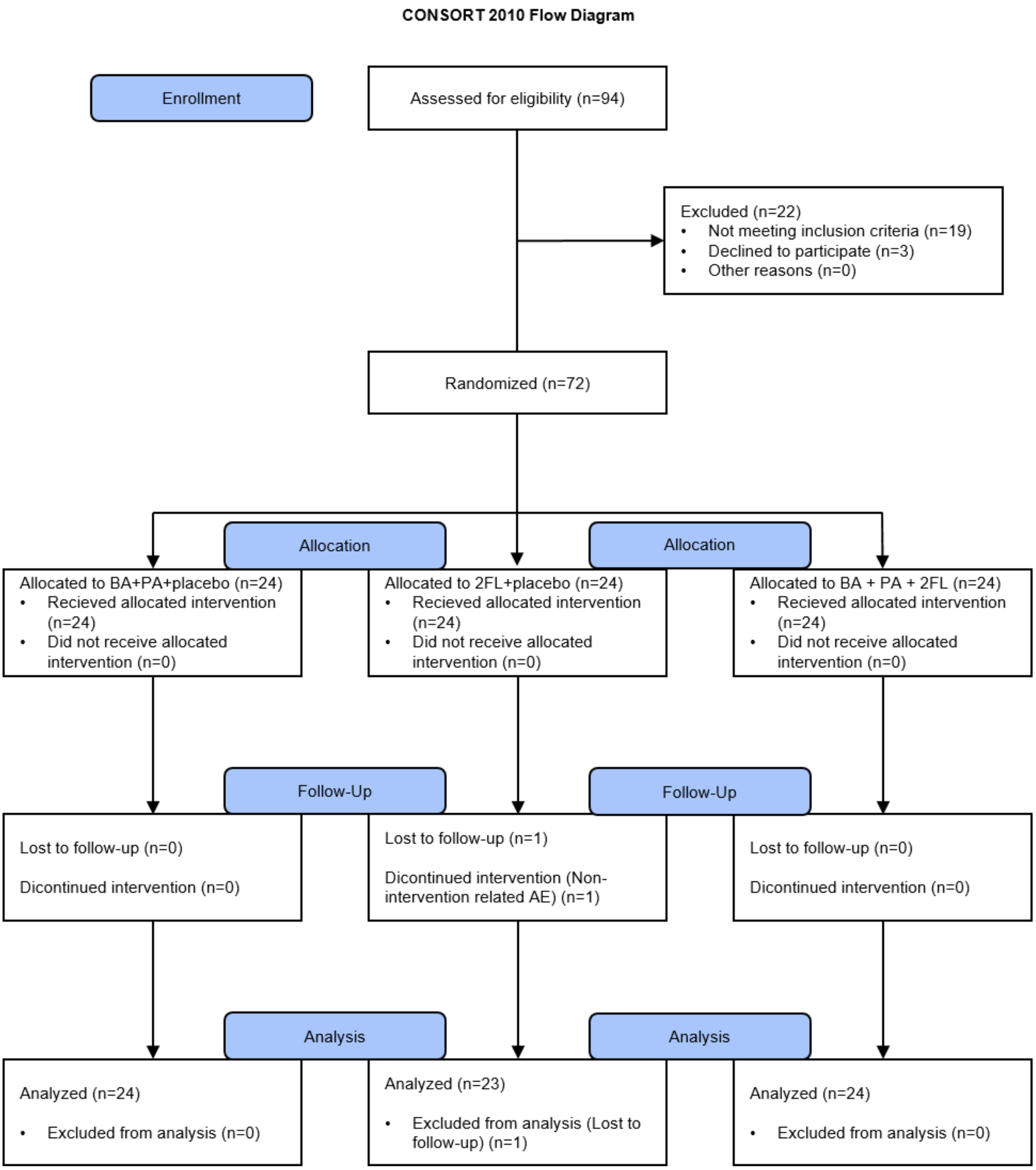

Supplementary Fig. S1. **CONSORT 2010 flow diagram.** CONsolidated Standards of Reporting Trials: Schematic representation of the randomized double-blind prospective study.

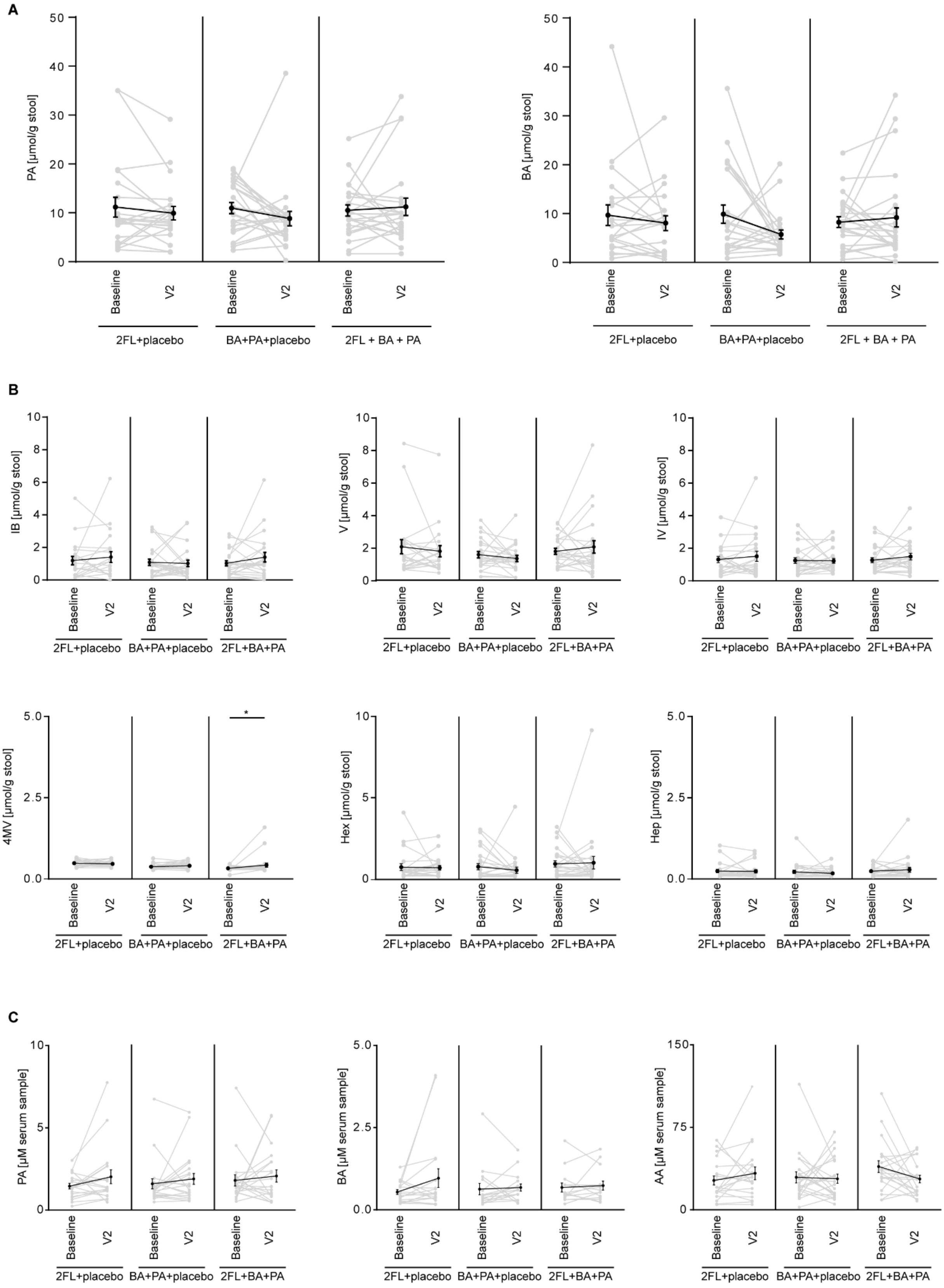

Supplementary Fig. S2. SCFA concentration in stool and serum is not altered after supplementation. (**A**) Concentrations of PA and BA and (**B**) various other SCFAs in stool

samples. IB = isobutyrate, V = valerate, IV = isovalerate, 4MV = 4-methyl-valerate, Hex = hexanoate, Hep = heptanoate. (**C**) PA, BA and acetic acid (AA) levels in serum samples. Concentrations measured by HPLC-MS/MS. Data are plotted as before-after for each patient (gray) and mean (black) +/- SEM. Tested by 2-way ANOVA with Sidak's multiple comparisons test; $^*p < 0.05$.

gating strategy panel 1

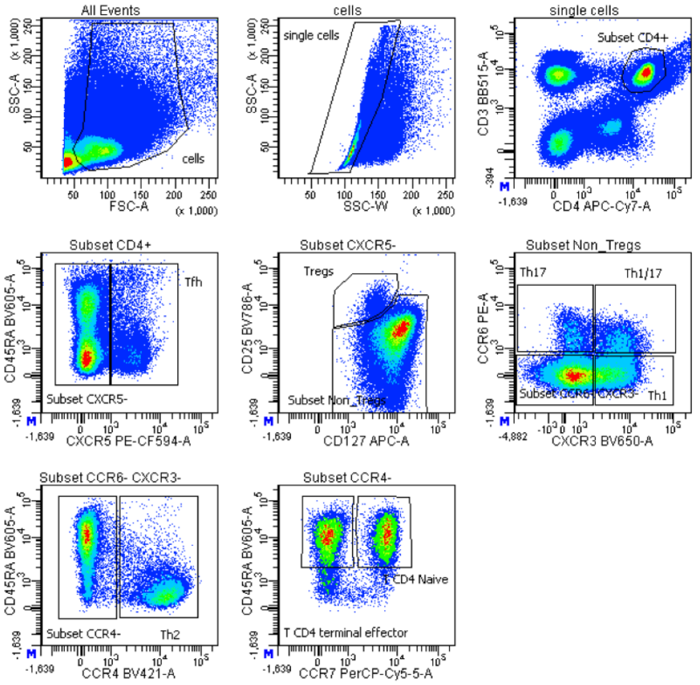

Well: G1 609 V2

Population	#Events	%Parent
All Events	320,028	####
cells	197,418	61.7
single cells	175,290	88.8
Subset CD4+	57,887	33.0
Tfh	8,006	13.8
Subset CXCR5-	49,566	85.6
Tregs	1,864	3.8
Subset Non_Tregs	47,334	95.5
Th17	3,057	6.5
Th1/17	3,729	7.9
Th1	12,582	26.6
Subset CCR6- CXCR3-	26,601	56.2
Th2	9,216	34.6
Subset CCR4-	17,277	64.9
T CD4 terminal effector	7,860	45.5
T CD4 Naive	7,328	42.4

gating strategy panel 2

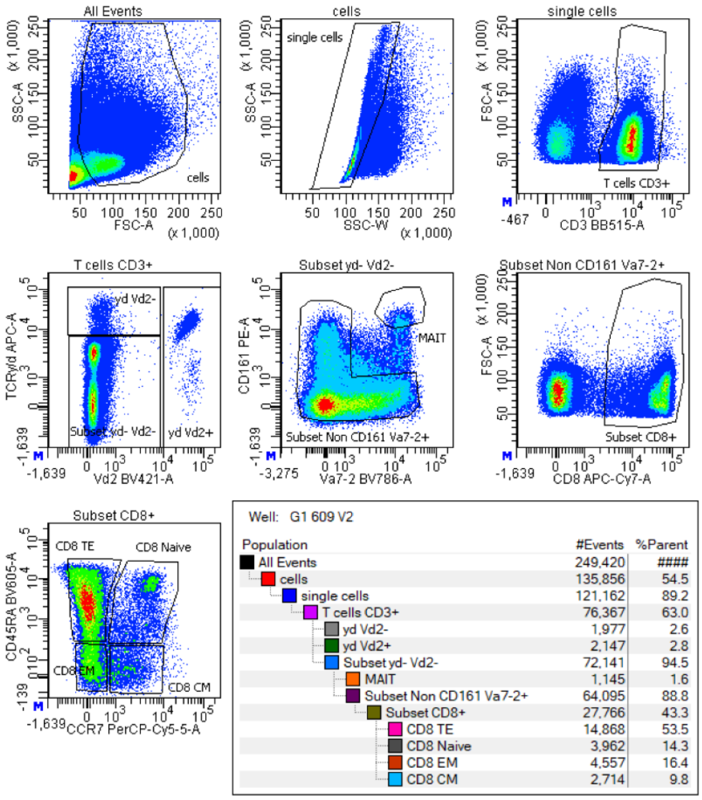

Well: G1 609 V2

Population	#Events	%Parent
All Events	249,420	####
cells	135,856	54.5
single cells	121,162	89.2
T cells CD3+	76,367	63.0
γd Vd2-	1,977	2.6
γd Vd2+	2,147	2.8
Subset γd- Vd2-	72,141	94.5
MAIT	1,145	1.6
Subset Non CD161 Va7-2+	64,095	88.8
Subset CD8+	27,766	43.3
CD8 TE	14,868	53.5
CD8 Naive	3,962	14.3
CD8 EM	4,557	16.4
CD8 CM	2,714	9.8

Supplementary Fig. S3: Gating strategy for flow cytometry analysis of cryopreserved PBMCs. Gating hierarchy for panel 1 (CD4 T cells). Samples passed quality control:

2FL+placebo: n = 14, BA+PA+placebo: n = 15, 2FL+BA+PA: n = 17; matched baseline and V2 samples. Gating hierarchy for panel 2 (conventional CD8 T cells, γδTCR+ and MAIT cells). Samples passed quality control: 2FL+placebo: n = 11, BA+PA+placebo: n = 12, 2FL+BA+PA: n = 17; matched baseline and V2 samples. All phenotyping experiments were performed on a BD FACS Celesta™ and analyzed by BD FACS DIVA v9 software.

gating strategy panel 3

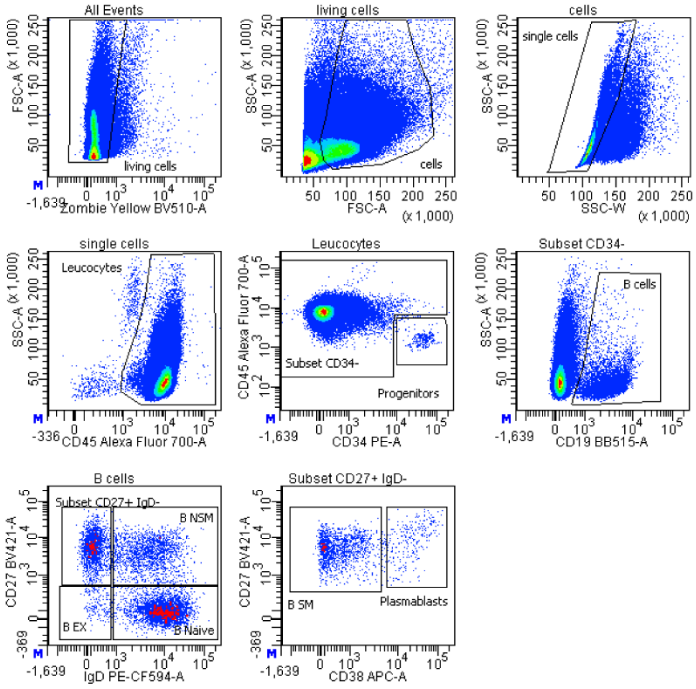

Well: G1 609 V2

Population	#Events	%Parent
All Events	320,749	####
living cells	316,996	98.8
cells	159,258	50.2
single cells	141,278	88.7
Leucocytes	140,605	99.5
Progenitors	245	0.2
Subset CD34-	140,329	99.8
B cells	6,909	4.9
B Naive	3,158	45.7
B NSM	1,725	25.0
B EX	168	2.4
Subset CD27+ IgD-	1,807	26.2
B SM	1,537	85.1
Plasmablasts	248	13.7

gating strategy panel 4

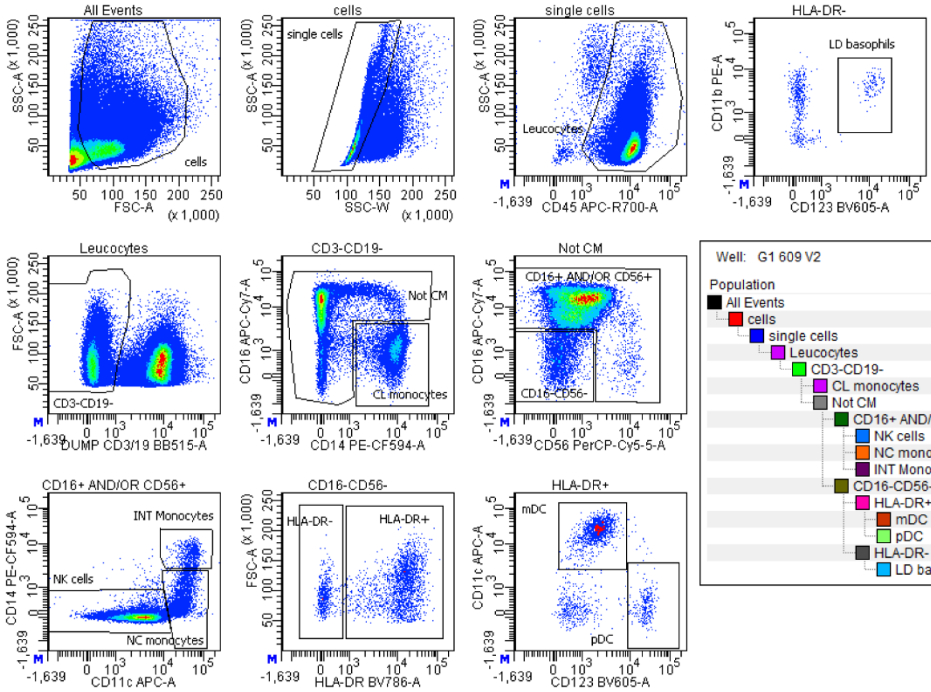

Well: G1 609 V2

Population	#Events	%Parent
All Events	219,954	####
cells	117,310	53.3
single cells	103,651	88.4
Leucocytes	102,384	98.8
CD3-CD19-	31,469	30.7
CL monocytes	6,270	19.9
Not CM	25,080	79.7
CD16+ AND/OR CD56-	22,285	88.9
NK cells	18,917	84.9
NC monocytes	2,486	11.2
INT Monocytes	711	3.2
CD16-CD56-	2,595	10.3
HLA-DR+	1,998	77.0
mDC	1,338	67.0
pDC	272	13.6
HLA-DR-	583	22.5
LD basophils	107	18.4

Supplementary Fig. S4: Gating strategy for flow cytometry analysis of cryopreserved PBMCs. Gating hierarchy for panel 3 (B cells and myeloid progenitors). Samples passed quality control: 2FL+placebo: n = 14, BA+PA+placebo: n = 15, 2FL+BA+PA: n = 17, matched baseline and V2 samples. Gating hierarchy for panel 4 (monocytes, dendritic cells, low density basophils and NK cells). Samples passed quality control: 2FL+placebo: n = 9, BA+PA+placebo: n = 10, 2FL+BA+PA: n = 13; matched baseline and V2 samples. All phenotyping experiments were performed on a BD FACS Celesta™ and analyzed by BD FACS DIVA v9 software.

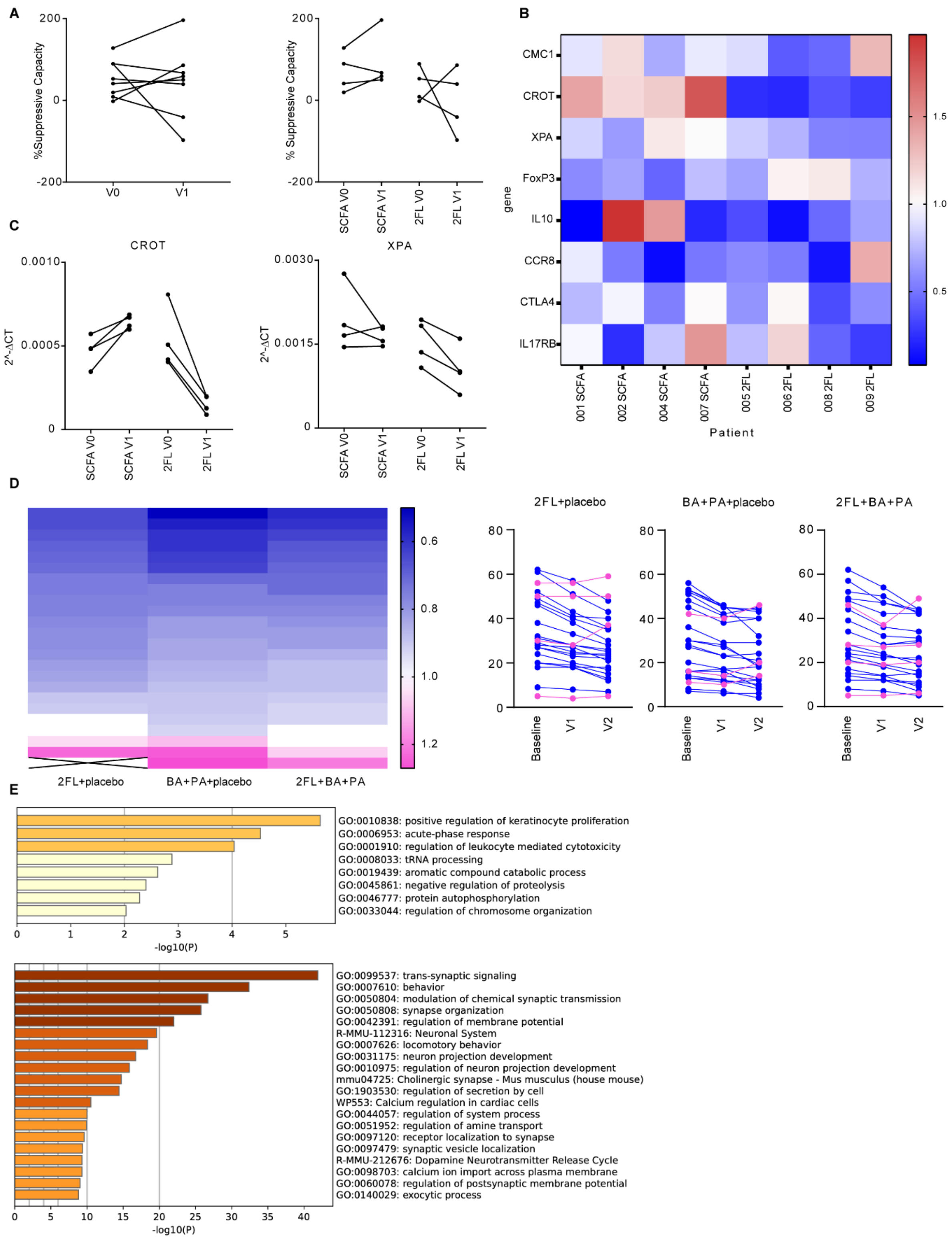

Supplementary Fig. S5: Supplementation with SCFA does not boost the suppressive capacity of Tregs but increases the expression of mitochondrial genes and clinical response to supplementation is associated with distinct *ex vivo* colonic responses. (A) Treg/PBMC suppression assay at baseline (V0) and 14-day follow-up (V1), samples from

validation cohort PrePD2, MACS-sorted cells. (**B, C**) Gene expression in sorted Tregs from validation cohort PrePD2. FACS-sorted cells. (B) Heatmap of fold changes follow-up (V1) and (C) individual 2^-ΔCT values for Crot and Xpa. Housekeeping B2m, time points compared by Wilcoxon matched pairs test. (**D**) Responders (R: MDS-UPDRS III V2 < MDS-UPDRS III baseline) and nonresponders (NR: MDS-UPDRS III V2 >/= MDS-UPDRS III baseline) stratified by group. Data plotted as heatmap (ratio V2/baseline) and MDS-UPDRS III before-after for each patient; responders (blue) and nonresponders (pink). (**E**) Gene Ontology analysis of transcripts enriched in gut cultures infused with R vs. NR microbiota using Metascape. Upper panel, pathways upregulated by R microbiota; lower panel, pathways induced by NR microbiota.

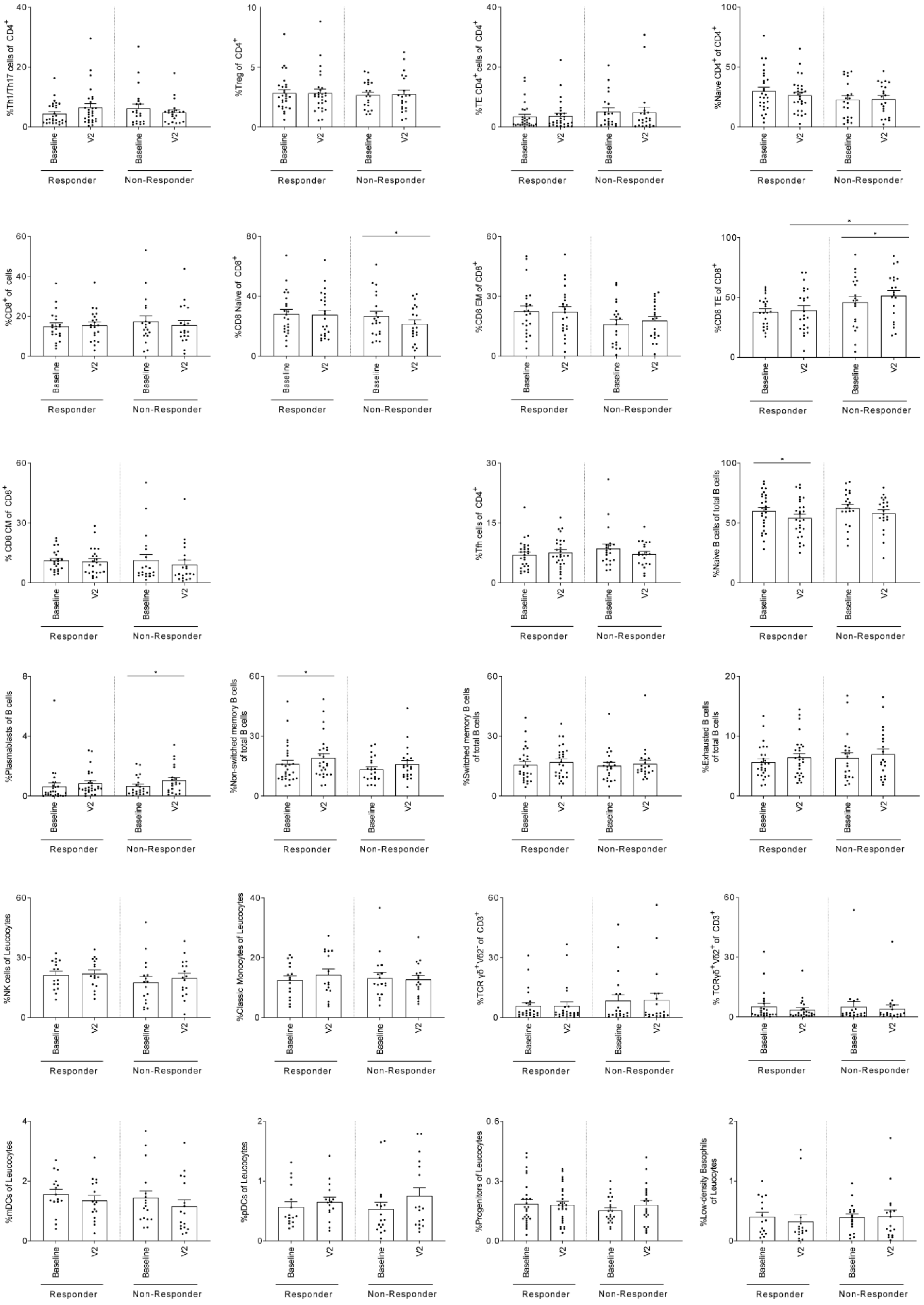

Supplementary Fig. S6: Supplementation with SCFAs and/or 2FL induces distinct changes in immune cell subsets in responding patients. In-depth immune profiling of frozen PBMCs at baseline and after 6 months of supplementation (V2). Proportions of

immune subsets; for gating strategies and antibodies, see Supplementary Figs. 3 and 4, Supplementary Tables and immunophenotyping in Methods section. All data are depicted as the mean ± SEM, median split responder/nonresponder according to MDS-UPDRS III. Paired T tests were performed for before-after comparisons, and unpaired T tests were performed for baseline and V2 R versus NR comparisons. $^{*}p < 0.05$.

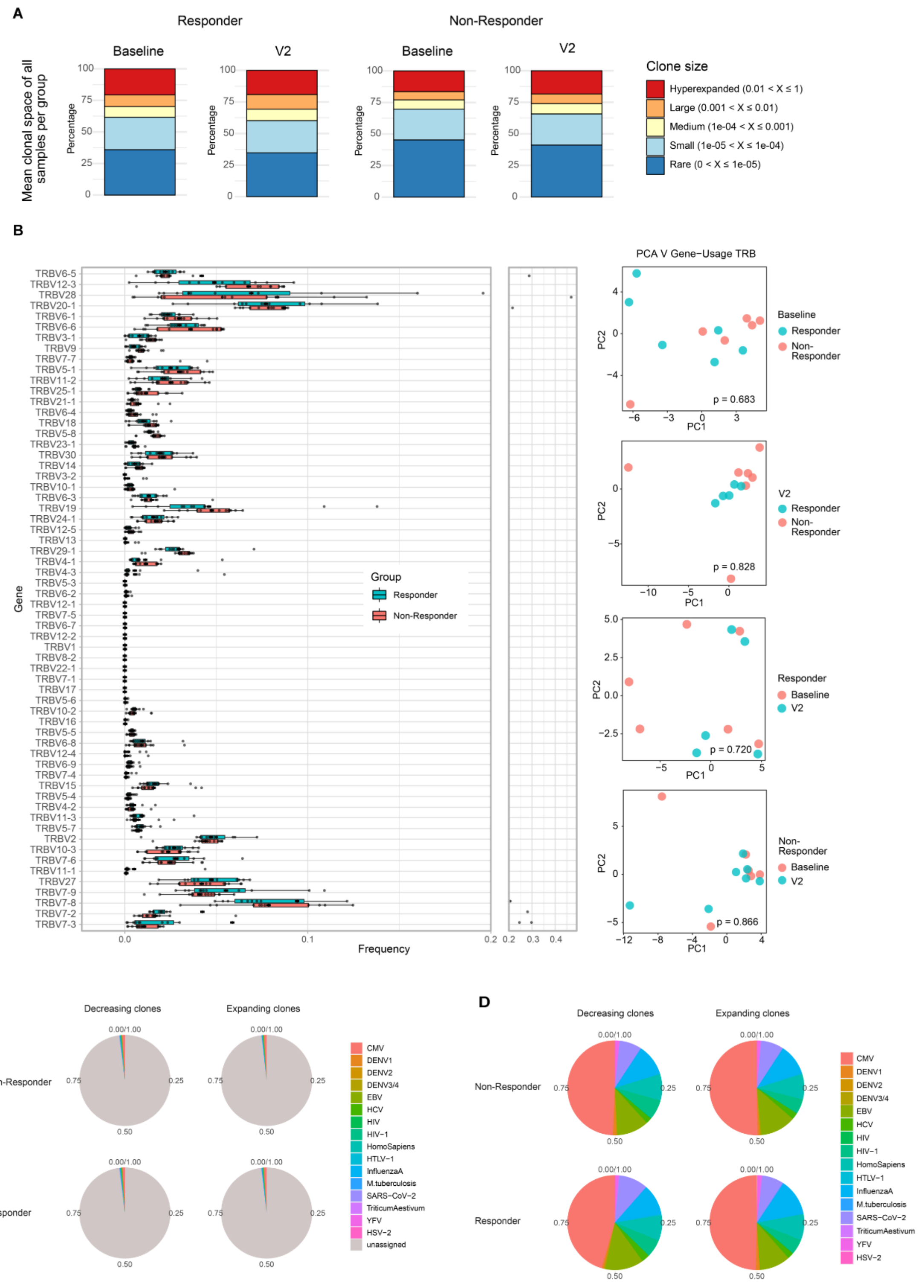

Supplementary Fig. S7: Extended analysis of TCRbeta repertoire characteristics and epitope recognition of TCRbeta clones. (**A**) Clonal space distribution for all samples per group. Percentage of repertoire taken up by hyperexpanded clones (frequency > 0.01) to rare clones (frequency < 0.00001) plotted as the mean per group. (**B**) TRBV gene usage within

repertoires of responders versus nonresponders (pooled baseline and V2). Each point refers to one repertoire. Boxes extend from 25% to 75% quartile with whiskers spanning 1.5* interquartile range; the bold line indicates the mean. Principal component analyses (PCA) of TRBV usage shown for the first and second dimension of each comparison. Small distances between points refer to similar TRBV gene usage, while long distances between samples reflect more different TRBV gene usage. P values were calculated with multivariate analysis of variance (MANOVA) according to Pillai. (**C, D**) Clones with decreased or expanded frequencies in responders or nonresponders (paired samples) upon supplementation were identified using the public database VDJdb30 (accessed 2nd August 2022) for matching CDR3 amino acids. Epitopes of TCRbeta with known function plotted per group including (C) or excluding (D) unassigned sequences.
