# Supplementary material for: Supplementation with short-chain fatty acids and a prebiotic improves clinical outcome in Parkinson’s disease: a randomized double-blind prospective study
Source: Sci Rep. 2025 Dec 5;16:315. doi: 10.1038/s41598-025-29692-x (PMC12770440; doi:10.1038/s41598-025-29692-x)
Supplement: Supplementary file 9 — Supplementary Material 9 [file 41598_2025_29692_MOESM9_ESM.docx]

**ADDITIONAL FILES**

Additional file 1: Supplementary Tables S1-S5 (pdf)

Additional file 2: Supplementary Figures S1-S7 (pdf)

Additional file 3: Data file S1 (Excel): Differential gene expression analysis comparing early (4h) colonic transcriptional responses to *ex vivo* infusion of fecal samples from PD patients post- vs. pre supplementation.

Additional file 4: Data file S2 (Excel): GSEA identified pathways significantly (FDR<=0.05) up- or downregulated following gut stimulation with post- versus pre supplementation microbiota.

Additional file 5: Data file S3 (Excel): Raw counts table of taxonomic abundance identified from *in vitro* epithelial adhesion assay (epithelial-adhesive microbes identified by co-culture of Caco-2 cells with fecal samples collected pre- and post-SCFA intervention, followed by 16S sequencing).

Additional file 6: Data file S4 (Excel): Differential gene expression analysis comparing early (4h) colonic transcriptional responses to *ex vivo* infusion of fecal samples from responding (R) vs. non-responding (NR) patients.

Additional file 7: Data file S5 (Excel): GSEA identified pathways significantly (FDR<=0.05) up- or downregulated following gut stimulation with microbiota from responding (R) vs. non-responding (NR) patients.
